# Algorithm-Based Palliative Care in Patients With Cancer: A Cluster Randomized Clinical Trial

**DOI:** 10.1001/jamanetworkopen.2024.58576

**Published:** 2025-02-21

**Authors:** Ravi B. Parikh, William J. Ferrell, Yang Li, Jinbo Chen, Larry Bilbrey, Nicole Johnson, Jenna White, Ramy Sedhom, Natalie R. Dickson, Stephen Schleicher, Justin E. Bekelman, Sandhya Mudumbi

**Affiliations:** 1 Division of Hematology and Medical Oncology, Emory University School of Medicine, Atlanta, Georgia; 2 Winship Cancer Institute, Atlanta, Georgia; 3 Department of Medical Ethics and Health Policy, Perelman School of Medicine at the University of Pennsylvania, Philadelphia; 4 Penn Center for Cancer Care Innovation, Abramson Cancer Center, University of Pennsylvania, Philadelphia; 5 Department of Biostatistics, Epidemiology, and Informatics, Perelman School of Medicine at the University of Pennsylvania, Philadelphia; 6 Tennessee Oncology, Nashville

## Abstract

**Importance:**

Among patients with advanced solid malignant tumors, early specialty palliative care (PC) is guideline recommended, but strategies to increase PC access and effectiveness in community oncology are lacking.

**Objective:**

To test whether algorithm-based defaults with opting out and accountable justification embedded in the electronic health record (EHR) increase completed PC visits.

**Design, Setting, and Participants:**

This 2-arm cluster randomized clinical trial was conducted from November 1, 2022, to December 31, 2023. Eligible patients from 15 urban or rural clinics within a large community oncology network in Tennessee had advanced lung or noncolorectal gastrointestinal cancer and were identified by an automated EHR algorithm adapted from national guidelines. Data were analyzed between November 1, 2023, and March 4, 2024.

**Intervention:**

At sites randomized to control, clinicians received weekly reports detailing PC referral rates compared with peer clinicians (peer comparison) and referred patients to PC at their discretion. At sites randomized to intervention, clinicians also received default PC orders using the EHR. Clinicians who opted out of PC consultation were asked to provide justification (accountable justification). If clinicians did not opt out, a study coordinator contacted patients to introduce and schedule PC visits using a standardized, predefined script.

**Main Outcomes and Measures:**

The primary outcome was a completed PC consultation within 12 weeks of enrollment. Exploratory outcomes included quality of life, feeling heard and understood, and intensive end-of-life care. Outcomes were analyzed using clustered generalized linear and logistic regression models.

**Results:**

The trial enrolled 562 patients (mean [SD] age, 68.5 [10.1] years; 288 male [51.2%]), of whom 433 (77.0%) had lung cancer. There were 130 of 296 patients (43.9%) randomized to the intervention group and 22 of 266 (8.3%) randomized to the control group who completed PC visits (adjusted odds ratio, 8.9 [95% CI, 5.5-14.6]; *P* < .001). Among 179 patients who died at the 24-week follow-up, 6 of 92 (6.5%) in the intervention group compared with 14 of 87 (16.1%) in the control group received systemic therapy within 14 days of death (adjusted odds ratio, 0.3 [95% CI, 0.1-0.7]; *P* = .05). There were no differences in quality of life, feeling heard and understood, or late hospice referral.

**Conclusions and Relevance:**

In this randomized clinical trial of algorithm-based EHR defaults, the intervention increased PC consultations and decreased end-of-life systemic therapy. The intervention provides a scalable implementation strategy to increase specialty PC referrals in the community oncology setting.

**Trial Registration:**

ClinicalTrials.gov Identifier: NCT05590962

## Key Points

**Question:**

Do default specialty palliative care orders with opting out and accountable justification increase consultation and improve outcomes in patients with cancer in an outpatient setting?

**Findings:**

In this randomized clinical trial conducted in a community oncology network between November 2022 and December 2023 among 562 patients with advanced cancer identified by an automated electronic health record algorithm, default orders increased palliative care consultation (44% vs 8%) and decreased end-of-life systemic therapy (6% vs 16%) compared with usual care but did not improve patient-reported or hospice outcomes.

**Meaning:**

The findings suggest that default algorithm-based palliative care orders are a scalable implementation strategy to increase palliative care referrals and reduce intensive end-of-life care.

## Teaser

This randomized clinical trial assessed whether algorithm-based default palliative care orders with opting out and accountable justification embedded in the electronic health record increase completed palliative care visits.

## Introduction

Early outpatient specialty palliative care (PC), concurrent with disease-directed cancer care, improves quality of life, decreases intensive end-of-life care, and is an evidence-based practice endorsed by national guidelines.^1,2,3,4,5,6,7,8^ Prior randomized clinical trials of early PC in oncology used highly controlled designs, primarily in academic settings, with interventions emphasizing frequent PC visits within weeks of an advanced diagnosis.^1,2,3,4,5,9^ There has been little experimental evidence of effectiveness of outpatient specialist PC in community oncology, in which 85% of patients receive their primary cancer care.^10,11^

Medical oncologists are often gatekeepers of outpatient PC referral for patients with advanced cancer.^12^ To refer a patient to PC, an oncology clinician must identify an appropriate patient, communicate the rationale for referral with the patient and/or caregiver, and place a referral using the electronic health record (EHR). Barriers throughout this process include clinician beliefs that PC is only appropriate late in the care trajectory; concerns about how patients and caregivers might receive this information; and difficulty identifying patients who would benefit from PC most urgently.^12,13,14^ Ongoing PC shortages necessitate strategies to triage referral based on patient need.^15,16,17,18^ This has led to recent efforts using algorithms to allocate PC consultations.^19^

Advances in EHR infrastructure allow automated specialty PC defaults—pre-set actions that take effect if a clinician does not opt out.^20,21^ Defaults may be enhanced with accountable justification, which requests explanations when clinicians opt out.^22^ We developed an intervention of risk algorithm-based default PC referrals with accountable justification, embedded within the EHR, to overcome barriers to PC referral while optimizing resources and staffing in a community oncology setting. In this randomized clinical trial, we tested the hypotheses that risk algorithm-based defaults would increase rates of initial specialty PC consultations and improve patient-reported quality-of-life and end-of-life outcomes in patients with advanced cancers.

## Methods

### Study Design

Between November 1, 2022, and December 31, 2023, we conducted a cluster randomized clinical trial comparing usual care with an algorithm-based default PC intervention among adults with advanced lung (small cell or non–small cell lung cancer) and noncolorectal gastrointestinal (GI) malignant tumors. Patients received care within a large community oncology network, encompassing rural and urban areas in Tennessee, that cares for an estimated 37% of Tennessee residents with cancer (eFigure 1 in Supplement 1). The protocol was approved by institutional review boards at the University of Pennsylvania and Advarra Inc, with a waiver of informed consent because this study was an evaluation of a health system initiative that posed minimal risk to clinicians and patients. The trial protocol is provided in Supplement 2. The study followed the Consolidated Standards of Reporting Trials (CONSORT) reporting guideline for cluster randomized clinical trials.

### Trial Sites and Patients

The trial was conducted in 15 community medical oncology clinics that all used the same EHR and had access to either an on-site or a virtual specialty PC clinician (eTable 1 in Supplement 1). We educated all sites about the trial prior to randomization but did not require clinics to change or refine their PC offerings or practices.

Patients 18 years or older were eligible for inclusion if they were actively receiving or had received care at the eligible clinic, had stage III or IV lung or noncolorectal GI malignant tumors, and were eligible for PC as determined by an automated EHR algorithm. Patients were not eligible if they were enrolled in hospice care; did not have a scheduled medical oncology follow-up appointment; had not had a medical oncology visit in the prior 24 weeks; had no prior EHR data; or only had scheduled visits for benign hematology, genetics, or survivorship. Patients who had previously seen PC were eligible if the visit was not within the past 90 days. Patients could be enrolled at any point in their disease course as long as they met algorithm criteria.

Patient age, sex, and race and ethnicity were identified from the EHR, which was primarily self-reported. Race and ethnicity categories included American Indian or Alaska Native, Asian, Black or African American, Hispanic or Latino, non-Hispanic or non-Latino, White, and other race or ethnicity (which included unknown and unspecified); these data were collected because of potential associations between non-White race and lower rates of palliative care referral.

### Algorithm

Individuals eligible for PC were identified using an EHR algorithm that assigned scores to each patient based on the number of PC risk factors met by that patient. The algorithm was adapted from the 2022 National Comprehensive Cancer Network palliative care guidelines (eTables 2 and 3 and eMethods in Supplement 1).^7^ The algorithm assigned a weighted score to each patient that reflected individual need for specialty PC consultation. We defined patients as PC eligible with a score of 1 or more for stage IV cancers and 2 or more for stage III cancers. Details on algorithm development and code mapping are available in the eMethods in Supplement 1.

### Randomization, Blinding, and Study Procedures

We chose a cluster randomized design, randomized at the clinic level to avoid contamination, as most clinicians practiced at only 1 clinic. We stratified randomization by clinic volume to avoid the possibility of large clinics being disproportionately randomized to the intervention (eTable 1 in Supplement 1 provides clinic grouping, and the eMethods in Supplement 1 provide a description of randomization). It was not feasible for participating oncology or PC clinicians to be blinded due to the nature of the intervention. The principal investigator (R.B.P.), faculty biostatistician (J.C.), and lead statistical analyst (Y.L.) were blinded to the allocation of clinics to intervention or control groups and to all outcomes until follow-up was completed.

To identify eligible patients in both arms, each Sunday, the EHR algorithm automatically identified patients with encounters in the following week who met prespecified score thresholds. Following identification of high-risk patients, research staff (N.J.) performed a brief chart review to confirm that the score was accurate and that the patient was not deceased, admitted to hospice care, assigned to an incorrect clinic, or already scheduled for a PC visit.

In both control and intervention clinics, physicians received performance reports detailing their individual rates of PC referral in relation to peer clinicians (eFigure 2 in Supplement 1). Performance reports and peer comparisons had been in place for 2 years prior to the start of the trial. In the control arm, no educational or other interventions related to PC occurred before or during the trial.

In the intervention arm, oncologists and advance practice clinicians providing care for that patient received default automated notifications using the clinician’s EHR inbox explaining the rationale for PC referral and offering an opportunity to opt out of the referral by replying to the inbox message (eFigure 3 in Supplement 1). If clinicians opted out, they were asked to reply to the EHR inbox notification with a reason for opting out of PC referral (accountable justification). If the clinician did not opt out within 48 hours, a nurse PC coordinator introduced themselves to the patient by telephone and offered a PC visit to the patient using a predefined script (eFigure 4 in Supplement 1). If a patient agreed to the consult, the coordinator scheduled the patient and triaged the urgency of the consult based on the algorithm score (eMethods in Supplement 1). Throughout the trial, clinicians in both arms could refer patients to PC at their discretion. The Figure describes the flow of patients and clinics in both arms.

**Figure.**
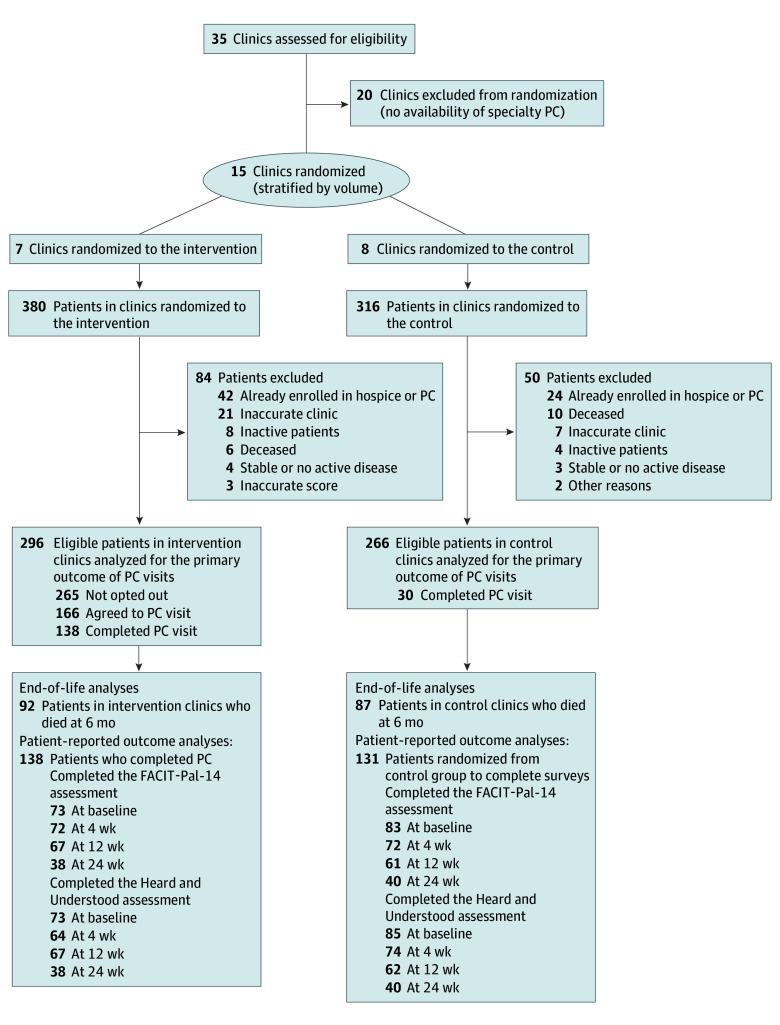
Patient and Clinic Flow Diagram Excluded patients were identified from chart review by research staff (N.J.) prior to offering clinician opt out. Inaccurate scores were identified if the automated algorithm mischaracterized 1 or more variables as present when they were actually absent by chart review. This resulted in patients not being high risk and thus ineligible. Patients were defined as inactive if, after randomization, they did not have a scheduled follow-up in any eligible clinics; inactive patients were not included in the analysis because it could not be confirmed that they saw palliative care (PC) externally. FACIT-Pal-14 indicates Functional Assessment of Chronic Illness Therapy-PC.

### Outcomes

All outcomes were defined at the patient level and extracted from the EHR. Follow-up was 24 weeks from enrollment. The primary outcome was a completed PC visit, measured as completion of an initial specialty PC consultation within 12 weeks of enrollment. PC visits conducted in person or using telemedicine within any of the 15 community oncology clinics were included in the outcome; canceled or no-show visits were not included. We chose completed PC visits as the outcome because our intervention aimed to change patterns and behaviors surrounding PC referrals rather than directly alter downstream effects, such as symptom burden.^23^

Exploratory outcomes included patient quality of life, feeling heard and understood, and markers of intensive end-of-life care. Patient quality of life was measured using the Functional Assessment of Chronic Illness Therapy-PC (FACIT-Pal-14) scale, a 14-item questionnaire that measures health-related quality of life overall (scores range from 0 to 56, with higher scores indicating better quality of life).^24,25^ Feeling heard and understood was measured using the 4-item Heard and Understood questionnaire, a validated measure of patient-centric communication, in which scores range from 0 to 4, with higher scores indicating a greater likelihood of feeling heard and understood.^26,27,28^ Patients were consented for quality-of-life assessments, and instruments were assessed by telephone (the eMethods in Supplement 1 provides a description of recruitment for and administration of surveys). Absolute change in FACIT-Pal-14 and Heard and Understood questionnaire scores between baseline and 24 weeks (4-week and 12-week comparisons as exploratory) were compared between intervention and control arms.

Intensive end-of-life care metrics were selected based on measures endorsed by national guidelines and used as performance metrics in alternative payment models and included receipt of systemic therapy (chemotherapy, immunotherapy, and/or targeted therapy) in the last 14 days of life and late hospice referral (hospice referral <3 days before death).^8,29^ These were measured among patients who died during trial follow-up.

### Statistical Analysis

Our primary analysis was modified intention to treat so that all patients who were eligible were included regardless of whether they received the intervention. A sample of 360 patients provided 80% power to detect a clinically meaningful 17 percentage-point improvement in the primary outcome of completed PC visits (from 27% to 44%), using a 2-sided type 1 error rate of 5%, 10% dropout rate, and intracluster correlation coefficient of 0.07. Trial enrollment was stipulated to stop at the latter of enrollment of 360 patients or 24 weeks after trial start.

Our primary analysis used adjusted generalized linear mixed models to assess the primary outcome. To account for death before completion of follow-up, in a secondary analysis, we used adjusted Cox proportional hazards regression models and Kaplan-Meier survival curves to analyze the primary outcome, with death as a censored event to account for differential mortality. All analyses included a binary indicator for intervention compared with control and adjusted for the following covariates assumed to influence receipt of PC: age, sex, race and ethnicity, diagnosis, stage, and risk score. We accounted for patient clustering by clinic using robust SE estimates. We report intervention effects in adjusted odds ratios (AORs) and adjusted hazard ratios, with higher ratios indicating greater rates of PC in the intervention arm. To analyze heterogeneity of treatment effects, we repeated analyses in 6 prespecified subgroups and used intervention-by-subgroup interaction terms.

To analyze end-of-life outcomes among decedents, we used logistic regression models, adjusted for all covariates and using robust SEs. To analyze patient-reported outcomes (quality of life and feeling heard and understood), we used linear regression with clustered SEs, adjusted for all covariates to measure the primary outcome at 24 weeks, as well as survey outcomes at the 4-week and 12-week time points. To account for survey noncompletion due to death, we used inverse probability of censoring weighting to weight all patient-reported outcomes by patient mortality risk.^30^ For primary analyses, we also adapted previous methods by carrying the most recent nonbaseline survey values forward to account for all missing patient-reported outcome data, excluding data that were missing due to death.^1^ To obtain 95% CIs for the survey analyses, we used the bootstrap method, resampling patients 1000 times and maintaining clustering at the clinic level. In a sensitivity analysis, we did not use the carry-forward method to analyze patient-reported metrics. All analyses were conducted between November 1, 2023, and March 4, 2024, using R, version 4.2.3 (R Project for Statistical Computing), with 2-sided *P* = .05 indicating statistical significance.

## Results

### Sample Characteristics and Covariates

Of 562 eligible patients in the modified intention-to-treat population across 15 clinics (mean [SD] age, 68.5 [10.1] years; 274 female [48.8%] and 288 male [51.2%]), 8 clinics representing 266 patients (47.3%) were randomized to the control arm, and 7 clinics representing 296 patients (52.7%) were randomized to the intervention arm (Table 1). Of the 562 randomized patients, 433 (77.0%) had lung cancer. Of these patients, 2 (0.4%) were American Indian or Alaska Native, 10 (1.8%) were Asian, 67 (11.9%) were Black or African American, 9 (1.6%) were Hispanic or Latino, 428 (76.2%) were non-Hispanic or non-Latino, 447 (79.5%) were White, 36 (6.4%) were of other race, and 125 (22.2%) were of other ethnicity. The median risk score was 2.0 (range, 1.0-20.0) in intervention arms and 2.0 (range, 1.0-18.0) in control arms. Among 296 intervention patients, 265 (89.5%) were not opted out of PC by their clinician; of those 265 patients, 166 (62.6%) agreed to PC (Figure). The most reported reasons for clinician opt out were patient symptom burden or performance status not severe enough to justify PC referral (n = 12), patient-specific barriers to seeing PC (n = 7), and timing of PC being too early (n = 7) (eTable 4 in Supplement 1). There were no meaningful differences in characteristics of patients opted out compared with those not opted out by clinicians (eTable 5 in Supplement 1). Four patients offered reasons for declining a PC visit; these reasons included well-controlled symptoms (n = 2) and feeling overburdened with visits (n = 2) (eTable 6 in Supplement 1).

**Table 1.** Patient Characteristics

Characteristic	Patient group^a^		
	Control (n = 266)	Intervention (n = 296)	Total (N = 562)
Sex			
Female	128 (48.1)	146 (49.3)	274 (48.8)
Male	138 (51.9)	150 (50.7)	288 (51.2)
Age, mean (SD) [range], y	69.2 (9.7) [39.0-93.0]	67.9 (10.4) [30.0-88.0]	68.5 (10.1) [30.0-93.0]
Race			
American Indian or Alaska Native	1 (0.4)	1 (0.3)	2 (0.4)
Asian	2 (0.8)	8 (2.7)	10 (1.8)
Black or African American	30 (11.3)	37 (12.5)	67 (11.9)
White	215 (80.8)	232 (78.4)	447 (79.5)
Other^b^	18 (6.8)	18 (6.1)	36 (6.4)
Ethnicity			
Hispanic or Latino	4 (1.5)	5 (1.7)	9 (1.6)
Non-Hispanic or non-Latino	216 (81.2)	212 (71.6)	428 (76.2)
Other^b^	46 (17.3)	79 (26.7)	125 (22.2)
Malignant tumor			
Lung	202 (75.9)	231 (78.0)	433 (77.0)
Noncolorectal GI	64 (24.1)	65 (22.0)	129 (23.0)
Cancer stage			
III	93 (35.0)	90 (30.4)	183 (32.6)
IV	173 (65.0)	206 (69.6)	379 (67.4)
Risk score			
Mean (SD)	3.2 (2.6)	3.0 (2.8)	3.1 (2.7)
Median (range)	2.0 (1.0-18.0)	2.0 (1.0-20.0)	2.0 (1.0-20.0)

Abbreviation: GI, gastrointestinal.

^a^ Data are reported as No. (%) of patients unless otherwise indicated.

^b^ Includes unknown and unspecified.

### PC Outcomes

Unadjusted rates of completed PC visits in the modified intention-to-treat population were 43.9% (130 of 296 patients) in the intervention arm and 8.3% (22 of 266 patients) in the control arm. In adjusted analyses, the intervention was associated with an increase in completed PC visits (AOR, 8.9 [95% CI, 5.5-14.6]; *P* < .001) (Table 2). Among individuals completing PC visits during the study period, the median number of PC visits (intervention: 2.9 [range, 1.0-6.0] vs control: 3.4 [range, 1.0-7.0]) and percentage of individuals with more than 1 PC visit (intervention: 84.2% vs control: 89.3%) were similar between intervention and control patients. Heterogeneity analyses revealed that the intervention had a disproportionate effect among patients with lung compared with noncolorectal GI malignant tumors (AOR, 14.1 [95% CI, 7.1-27.9] vs 4.2 [95% CI, 1.8-9.9]; *P* = .03 for interaction). Secondary analyses using Cox proportional hazards regression models were consistent with the primary analysis (eTable 7 in Supplement 1).

**Table 2.** Adjusted Changes in PC Referral Rates^a^

Population	Unadjusted PC rates, %		AOR (95% CI)^b^	*P* value^c^
	Control group (n = 266)	Intervention group (n = 296)		
Overall	8.3	43.9	8.9 (5.5-14.6)	<.001
**Subgroup analyses**				
Sex				
Female	9.4	48.0	9.4 (4.6-19.3)	.91
Male	7.3	40.0	8.7 (4.2-17.9)	
Age				
≤70 y	10.3	46.6	7.9 (4.2-14.9)	.42
>70 y	5.8	40.7	12.1 (5.2-28.2)	
Race and ethnicity^d^				
Non-Hispanic White	9.4	41.3	7.1 (4.1-12.1)	.06
Other^e^	3.9	53.0	32.3 (6.7-155.0)	
Malignant tumor				
Lung	5.5	43.3	14.1 (7.1-27.9)	.03
Noncolorectal GI	17.2	46.2	4.2 (1.8-9.9)	
Cancer stage				
III	6.5	45.6	12.4 (4.7-32.5)	.43
IV	9.3	43.2	7.8 (4.3-14.1)	
Risk score				
≤3	7.8	43.6	9.7 (5.3-17.6)	.53
>3	9.5	44.9	8.0 (3.2-20.2)	

Abbreviations: AOR, adjusted odds ratio; GI, gastrointestinal; PC, palliative care.

^a^ All modified intention-to-treat analyses used generalized linear models, adjusting for patient age, sex, diagnosis, stage, and risk score using robust SEs to account for clustering by clinic.

^b^ AORs were derived for 12-week PC rates. AORs greater than 1 indicate a greater effect on PC for intervention relative to control.

^c^ The *P* value for the primary analysis in the overall cohort comes from the adjusted generalized linear models using α = .05 to define statistical significance. The *P* value for the 6 subgroup analyses reflects the significance of intervention-by-subgroup interaction terms using α = .05 to define statistical significance.

^d^ The race and ethnicity subgroup analysis was analyzed as White vs non-Hispanic White for interaction models to converge for more granular race and ethnicity categories due to low event rates in individual subgroups.

^e^ Includes American Indian or Alaska Native, Asian, Black or African American, Hispanic, unknown, and unspecified.

### End-of-Life Outcomes

Among 562 patients in the trial, 179 (31.9%) died (mortality rate: 92 of 296 [31.1%] in the intervention group and 87 of 266 [32.7%] in the control group; *P* = .44) within 24 weeks of enrollment; there was no difference in overall survival between arms (eFigure 5 in Supplement 1). Rates of systemic therapy within 14 days of death were 6.5% (6 of 92 patients) in the intervention group and 16.1% (14 of 87 patients) in the control group (AOR, 0.3 [95% CI, 0.1-0.7]; *P* = .05) (Table 3). There was no difference in late hospice referral between groups (9.8% [9 of 92 patients] in the intervention group vs 13.8% [12 of 87 patients] in the control group; AOR, 0.6 [95% CI, 0.3-1.1]; *P* = .36).

**Table 3.** End-of-Life Outcomes^a^

Outcome	Intervention group, % (n = 92)	Control group, % (n = 87)	AOR (95% CI)	*P* value
Systemic therapy in the last 14 d of life	6.5	16.1	0.3 (0.1-0.7)	.05
Hospice enrollment <3 d before death	9.8	13.8	0.6 (0.3-1.1)	.36

Abbreviation: AOR, adjusted odds ratio.

^a^ Adjusted modified intention-to-treat analyses for end-of-life outcomes used logistic regression models, adjusting for patient age, sex, diagnosis, stage, and risk score using robust SEs to account for clustering by clinic.

### Patient-Reported Outcomes

At baseline, 64.9% (85 of 131) of control patients and 52.9% (73 of 138) of intervention patients completed either the FACIT-Pal-14 or the Heard and Understood assessment. At 24 weeks, 47.1% (40 of 85) of control patients and 52.1% (38 of 73) of intervention patients completed any follow-up assessment. Adjusted analyses at 24 weeks did not show any significant differences between intervention and control arms in the change in FACIT-Pal-14 scores (mean difference, −0.5 [95% CI, −0.8 to −0.2]; *P* = .68) and Heard and Understood scores (mean difference, −0.1 [95% CI, −0.1 to −0.1]; *P* = .51) (Table 4). In sensitivity analyses that analyzed differences at 4 weeks and 12 weeks and did not use the carry-forward method, no significant intervention-associated effects in patient-reported metrics were observed (eTable 8 in Supplement 1).

**Table 4.** Adjusted Mean Changes in Patient Quality of Life and Feeling Heard and Understood^a^

Mean change in assessment	Patients who completed FACIT-Pal-14 and Heard and Understood assessments				Adjusted difference between intervention and control groups, mean (95% CI)^d^	*P* value
	Control group^b^		Intervention group^c^			
	Completed follow-up survey, No. (%)	Score difference, mean (SD)	Completed follow-up survey, No. (%)	Score difference, mean (SD)		
FACIT-Pal-14						
Between 0 and 4 wk	72 (86.7)	1.1 (5.8)	72 (98.6)	1.0 (5.7)	0.12 (−0.03 to 0.3)	.69
Between 0 and 12 wk	61 (73.5)	1.5 (6.6)	67 (91.8)	2.1 (7.2)	0.4 (0.2 to 0.6)	.68
Between 0 and 24 wk	40 (48.2)	1.4 (6.1)	38 (52.1)	1.2 (8.6)	−0.5 (−0.8 to −0.2)	.68
Heard and Understood^e^						
Between 0 and 4 wk	74 (87.1)	−0.1 (0.6)	64 (87.7)	−0.1 (0.5)	−0.1 (−0.1 to −0.02)	.76
Between 0 and 12 wk	62 (72.9)	−0.03 (0.6)	67 (91.8)	−0.05 (0.6)	−0.04 (−0.1 to 0)	.74
Between 0 and 24 wk	40 (47.1)	0 (0.7)	38 (52.1)	−0.1 (0.6)	−0.1 (−0.1 to −0.1)	.51

Abbreviation: FACIT-Pal-14, Functional Assessment of Chronic Illness Therapy-Palliative Care (scores range from 0 to 56, with higher scores indicating better quality of life).

^a^ All modified intention-to-treat analyses for patient-reported outcomes used linear regression models, adjusting for patient age, sex, diagnosis, stage, and risk score using clustered SEs to account for clustering by clinic.

^b^ In the control group, 83 patients completed the FACIT-Pal-14 baseline assessment, and 85 patients completed the Heard and Understood baseline assessment.

^c^ In the intervention group, 73 patients completed the FACIT-Pal-14 baseline assessment, and 73 patients completed the Heard and Understood baseline assessment.

^d^ Intervention effects were presented as absolute adjusted differences (intervention group minus control group). Positive differences indicate a greater increase in quality of life or feeling heard and understood between baseline and 24 weeks in the intervention, relative to control. To obtain 95% CIs, the bootstrap method was used, resampling patients 1000 times. Resampling patients maintained clustering at the clinic level.

^e^ A questionnaire in which scores range from 0 to 4, with higher scores indicating greater likelihood of feeling heard and understood.

## Discussion

In this cluster randomized clinical trial among patients with advanced solid malignant tumors in a community oncology network, algorithm-based default PC referrals with accountable justification increased completed PC visits from 8.3% to 43.9% and decreased end-of-life systemic therapy from 16.1% to 6.5%. No changes in late hospice referral, quality of life, or feeling heard and understood were observed. Prior efficacy trials in oncology have tested early PC in controlled, primarily academic settings.^1,2,3,4,5,31,32,33,34^ To our knowledge, this is the first effectiveness randomized clinical trial of algorithm-driven default specialty PC in community oncology.

First, our intervention resulted in a large increase in outpatient specialty PC visits among high-risk patients, showing the effectiveness of the algorithm-driven default referral process. Timely referral to specialty PC is a metric of high-quality supportive cancer care.^35,36^ However, despite 80% of community oncology clinics having access to outpatient specialty PC, timing and referral rates vary widely, with only 8% of eligible patients receiving outpatient specialty PC referral.^15,37^ Furthermore, lack of PC utilization and access is more pronounced in rural settings, where 2 of the clinics in this study were located.^16^ Our intervention represents a novel approach to triage and increased access to specialty PC for diverse populations in resource-constrained settings.

Second, even though the intervention led to a large increase in referrals among high-risk patients, overall PC volume only increased by approximately 100 patients above baseline during the trial. There were also promising signs of patient and clinician acceptability of our intervention, with only 10.5% of clinicians opting out of referrals and 37.4% of patients refusing referral. Trained nonphysician coordinators introducing PC using standardized scripts and accepted messaging, as in our intervention, may reduce patient discomfort with PC.^38,39^

Third, our intervention led to a decrease in systemic therapy at the end of life, which is a guideline-endorsed measure of low-value end-of-life care.^36,40^ The intervention was associated with a numerical decrease in late hospice referrals, but this did not reach statistical significance. While patient-level randomized clinical trials of early PC have shown larger decreases (approximately 20 percentage points) in aggressive end-of-life care,^1^ other trials have not shown an effect on intensive end-of-life care.^4^ Our intervention showed a larger effect (9.6 percentage points) on end-of-life systemic therapy use than did another randomized clinical trial of a clinician-directed, algorithm-driven default text-message intervention to prompt earlier serious illness communication (2.9 percentage points).^41,42^ Clinician-directed defaults may have the strongest effect on metrics that are most directly within an oncologist’s control (such as the decision to order chemotherapy) than timing of hospice referral, which is influenced by other factors such as local availability of home-based hospice and hospitals’ referral patterns.^43,44^

Fourth, our intervention did not improve quality of life or feeling heard and understood. This stands in contrast to prior efficacy randomized clinical trials of early PC, which have shown benefits on quality of life.^1,3,45^ There are several factors that may explain this. As in other PC trials in community oncology settings, there was an approximate 50% rate of missing patient-reported outcome assessments at 24 weeks (47.1% of control patients and 52.1% of intervention patients), unlike the less than 30% noncompletion rates seen in PC efficacy trials in academic settings.^1,3,5,46,47^ This rate of survey nonresponse is somewhat expected given that the mortality rate in our trial was 31.9%, that other PC trials have shown 50% survey attrition rates at 6 months,^4^ and that attrition rates in pragmatic trials (ie, trials that include representative patients and clinicians) may be higher than typical randomized clinical trials. Additionally, among those who completed at least 1 PC visit, the majority of patients in our cohort had more than 1 PC visit (84.2% in the intervention group and 89.3% in the control group), and the median number of PC visits was 2.9 (range, 1.0-6.0) in the intervention group and 3.4 (range, 1.0-7.0) in the control group, whereas patients in prior efficacy trials had up to 6 visits during study follow-up.^5,48^ It is possible that follow-up PC visits occurred after the 6-month follow-up period, and even a single PC consultation may provide anticipatory guidance and symptom benefit. Algorithm-based interventions like ours may synergize with stepped models that intensify PC visits based on patient-reported quality of life.^49^

Defaults address key clinician barriers to specialty PC referral, including misconceptions of PC as incompatible with cancer therapy.^50,51^ While defaults have been useful for other high-value oncology care clinics, our trial suggests that defaults can also improve the timing and frequency of guideline-recommended PC consultation.^52^

### Limitations

This study has several limitations. First, while we studied effectiveness across a large oncology network, the findings in this single network may not be generalizable to other community-based systems without embedded PC.^15^ Second, our PC algorithms were unable to incorporate patient-reported variables beyond depression and distress screening surveys and, while based on national guidelines, had not been externally validated. Our algorithm may have missed patients with elevated symptoms, psychosocial conditions, or caregiver burden, who did not meet other criteria. Third, our patient-reported outcome findings should be interpreted with caution, as they may have been underpowered due to low completion of assessments. Furthermore, we sought to measure the direct effect of PC on patient-reported outcomes by comparing patients who received PC in the intervention group against a random subset of patients in the control group; thus, patient-reported outcome findings cannot be interpreted as a modified intention-to-treat comparison.

## Conclusions

In this randomized clinical trial, an intervention combining algorithm-based automated identification of patients eligible for PC with default PC referral led to an increase in PC visits and a decrease in end-of-life systemic therapy among patients with cancer. This study provides guidance for scalable, algorithm-driven PC implementation across community oncology settings.

## Notes

Supplement 1. **eFigure 1.** Map of Clinic Locations **eTable 1.** Clinic Locations **eFigure 2.** Peer Comparison Example **eTable 2.** Palliative Care Eligibility Algorithm **eTable 3.** Code Definitions for Eligible Patients and Algorithm Mapping **eFigure 3.** Sample EHR Inbox Message Sent for Intervention Patients **eFigure 4.** Palliative Care Script **eTable 4.** Reasons for Clinician Opt Out **eTable 5.** Characteristics of Patients Opted Out vs Not Opted Out in the Intervention Arm **eTable 6.** Reasons Cited by Patients for Declining Palliative Care Referral **eTable 7.** Adjusted Changes in Palliative Care Referral Rates in Secondary Analyses **eTable 8.** Adjusted Changes in Patient-Reported Metrics Not Using the Carry-Forward Method **eFigure 5.** Overall Survival Kaplan-Meier Plot
eMethods

Supplement 2.
Trial Protocol

Supplement 3.
Data Sharing Statement
