# Supplementary material for: Algorithm-Based Palliative Care in Patients With Cancer: A Cluster Randomized Clinical Trial
Source: JAMA Netw Open. 2025 Feb 21;8(2):e2458576. doi: 10.1001/jamanetworkopen.2024.58576 (PMC11846008; doi:10.1001/jamanetworkopen.2024.58576)
Supplement: Supplement 1. — eFigure 1. Map of Clinic Locations eTable 1. Clinic Locations eFigure 2. Peer Comparison Example eTable 2. Palliative Care Eligibility Algorithm eTable 3. Code Definitions for Eligible Patients and Algorithm Mapping eFigure 3. Sample EHR Inbox Message Sent for Intervention Patients eFigure 4. Palliative Care Script eTable 4. Reasons for Clinician Opt Out eTable 5. Characteristics of Patients Opted Out vs Not Opted Out in the Intervention Arm eTable 6. Reasons Cited by Patients for Declining Palliative Care Referral eTable 7. Adjusted Changes in Palliative Care Referral Rates in Secondary Analyses eTable 8. Adjusted Changes in Patient-Reported Metrics Not Using the Carry-Forward Method eFigure 5. Overall Survival Kaplan-Meier Plot eMethods [file jamanetwopen-e2458576-s001.pdf]

Supplemental Online Content

Parikh RB, Ferrell WJ, Li Y, et al. Algorithm-based palliative care in patients with cancer: a cluster randomized clinical trial. *JAMA Netw Open*. 2025;8(2):e2458576. doi:10.1001/jamanetworkopen.2024.58576

eFigure 1. Map of Clinic Locations
eTable 1. Clinic Locations
eFigure 2. Peer Comparison Example
eTable 2. Palliative Care Eligibility Algorithm
eTable 3. Code Definitions for Eligible Patients and Algorithm Mapping
eFigure 3. Sample EHR Inbox Message Sent for Intervention Patients
eFigure 4. Palliative Care Script
eTable 4. Reasons for Clinician Opt Out
eTable 5. Characteristics of Patients Opted Out vs Not Opted Out in the Intervention Arm
eTable 6. Reasons Cited by Patients for Declining Palliative Care Referral
eTable 7. Adjusted Changes in Palliative Care Referral Rates in Secondary Analyses
eTable 8. Adjusted Changes in Patient-Reported Metrics Not Using the Carry-Forward Method
eFigure 5. Overall Survival Kaplan-Meier Plot
eMethods

This supplemental material has been provided by the authors to give readers additional information about their work.

© 2025 Parikh RB et al. *JAMA Network Open*.

eFigure 1. Map of Clinic Locations

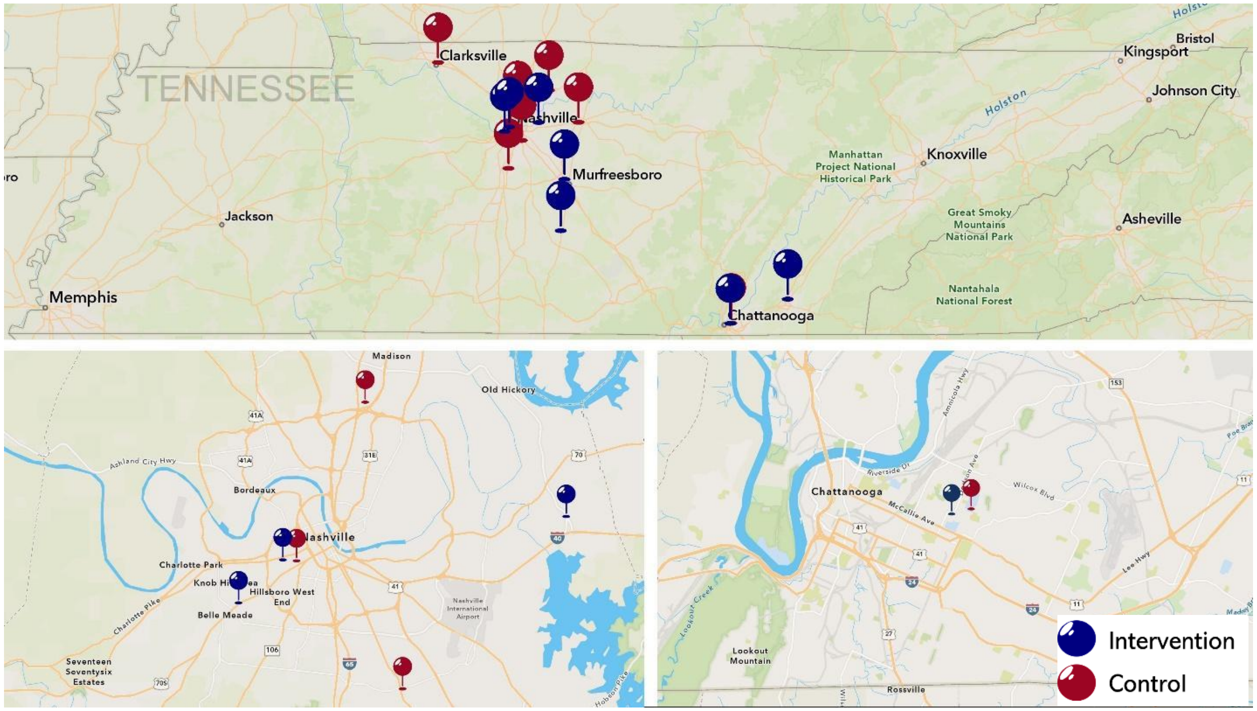

NOTE: Figure A shows the distribution of clinics included in the study across Tennessee. Given overlap of clinics in the Nashville (Figure B) and Chattanooga (Figure C) areas, the bottom panels show zoomed-in versions of these two cities.

© 2025 Parikh RB et al. *JAMA Network Open*.

eTable 1. Clinic Locations

Clinic location	Number of eligible patients in prior 6 months	Rural vs. urban	Intervention vs. control
Centennial	648	Urban	Intervention
Cleveland	248	Rural	Intervention
Medical Park II	453	Urban	Intervention
Murfreesboro	685	Urban	Intervention
Saint Thomas West	374	Urban	Intervention
Shelbyville	146	Rural	Intervention
Summit	276	Urban	Intervention
Clarksville	306	Urban	Control
Franklin	337	Urban	Control
Gallatin	293	Urban	Control
Lebanon	229	Urban	Control
Memorial	959	Urban	Control
Midtown	320	Urban	Control
Skyline	177	Urban	Control
Southern Hills	117	Urban	Control

© 2025 Parikh RB et al. *JAMA Network Open*.

eFigure 2. Peer Comparison Example

Physician Dashboard:

for time period 12/01/2021 - 12/31/2021

OneOncology

Below is a summary of your personal metrics and how you compare to your peers

Quality Metrics

	Your performance		*Your peers' performance YTD*	
	Last month	YTD	Practice Average	OO Average
**Staging Completion within 30 days of first visit**	70%	100%	83%	70%
**Via Pathway adherence rate**	94%	-	85%	88%
**Palliative care referals**				
Stage IV Lung	0%	-	33%	25%
Stage IV Pancreas	-	-	-	100%

NOTE: Peer comparisons were available for control and intervention practices and were in place 2 years prior to study start. Practice average referred to rates within Tennessee Oncology. OO average referred to averages within OneOncology, a broader network of value-based oncology practices within which Tennessee Oncology was part of.

© 2025 Parikh RB et al. *JAMA Network Open*.

eTable 2. Palliative Care Eligibility Algorithm

Criteria	Score	Source
Bone metastases	1	EHR diagnosis list[a]
Other distant metastases	1	EHR diagnosis list[a]
Chronic obstructive pulmonary disease	1	EHR diagnosis list[a]
Congestive heart failure	1	EHR diagnosis list[a]
Chronic kidney disease	1	EHR diagnosis list[a]
Dementia	1	EHR diagnosis list[a]
Spinal cord compression	1	EHR diagnosis list[a]
Most recent NCCN Distress Score ≥8	1	Structured EHR data
Most recent ECOG Performance Status ≥2	1	Structured EHR data
Most recent PHQ-2 Score≥3	1	Structured EHR data
Number of documented ED visits in prior 12 weeks	Each episode receives 2 points	EHR acute care utilization database
Number of documented inpatient stays in prior 12 weeks	Each inpatient stay receives 2 points	EHR acute care utilization database
Age ≤40	1	EHR Date of Birth
Age ≥70	1	EHR Date of Birth

NOTE: EHR = electronic health record; NCCN = National Comprehensive Cancer Network; ECOG = Eastern Cooperative Oncology Group; PHQ-2 = Patient Health Questionnaire-2.
[a]Diagnosis code mapping provided in eTable 3

© 2025 Parikh RB et al. *JAMA Network Open*.

eTable 3. Code Definitions for Eligible Patients and Algorithm Mapping

Cancer	ICD-10 codes
Non-small cell lung cancer	C34.*
Non-colorectal gastrointestinal cancer	C23.*, C24.*, C15.*, C16.*, C25.*
**Codes used to determine metastatic status (Table 1)**	
Malignant neoplasm of liver, not specified as primary or secondary	155.2
Secondary malignant neoplasm of mediastinum	197.1
Secondary malignant neoplasm of pleura	197.2
Secondary malignant neoplasm of other respiratory organs	197.3
Secondary malignant neoplasm of small intestine including duodenum	197.4
Secondary malignant neoplasm of large intestine and rectum	197.5
Secondary malignant neoplasm of retroperitoneum and peritoneum	197.6
Malignant neoplasm of liver, secondary	197.7
Secondary malignant neoplasm of other digestive organs and spleen	197.8
Secondary malignant neoplasm of other urinary organs	198.1
Secondary malignant neoplasm of skin	198.2
Secondary malignant neoplasm of brain and spinal cord	198.3
Secondary malignant neoplasm of other parts of nervous system	198.4
Secondary malignant neoplasm of bone and bone marrow	198.5
Secondary malignant neoplasm of ovary	198.6
Secondary malignant neoplasm of adrenal gland	198.7
Secondary malignant neoplasm of breast	198.81
Secondary malignant neoplasm of genital organs	198.82
Secondary malignant neoplasm of other specified sites	198.89
Secondary neuroendocrine tumor of liver	209.72
Secondary neuroendocrine tumor of bone	209.73
Secondary neuroendocrine tumor of peritoneum	209.74
Secondary Merkel cell carcinoma	209.75
Secondary neuroendocrine tumor of other sites	209.79

© 2025 Parikh RB et al. *JAMA Network Open*.

Secondary malignant neoplasm of unspecified lung	C78.00
Secondary malignant neoplasm of right lung	C78.01
Secondary malignant neoplasm of left lung	C78.02
Secondary malignant neoplasm of mediastinum	C78.1
Secondary malignant neoplasm of pleura	C78.2
Secondary malignant neoplasm of unspecified respiratory organ	C78.30
Secondary malignant neoplasm of other respiratory organs	C78.39
Secondary malignant neoplasm of small intestine	C78.4
Secondary malignant neoplasm of large intestine and rectum	C78.5
Secondary malignant neoplasm of retroperitoneum and peritoneum	C78.6
Secondary malignant neoplasm of liver and intrahepatic bile duct	C78.7
Secondary malignant neoplasm of unspecified digestive organ	C78.80
Secondary malignant neoplasm of other digestive organs	C78.89
Secondary malignant neoplasm of unspecified kidney and renal pelvis	C79.00
Secondary malignant neoplasm of right kidney and renal pelvis	C79.01
Secondary malignant neoplasm of left kidney and renal pelvis	C79.02
Secondary malignant neoplasm of unspecified urinary organs	C79.10
Secondary malignant neoplasm of bladder	C79.11
Secondary malignant neoplasm of other urinary organs	C79.19
Secondary malignant neoplasm of skin	C79.2
Secondary malignant neoplasm of brain	C79.31
Secondary malignant neoplasm of cerebral meninges	C79.32
Secondary malignant neoplasm of unspecified part of nervous system	C79.40
Secondary malignant neoplasm of other parts of nervous system	C79.49
Secondary malignant neoplasm of bone	C79.51
Secondary malignant neoplasm of bone marrow	C79.52
Secondary malignant neoplasm of unspecified ovary	C79.60

© 2025 Parikh RB et al. *JAMA Network Open*.

Secondary malignant neoplasm of right ovary	C79.61
Secondary malignant neoplasm of left ovary	C79.62
Secondary malignant neoplasm of unspecified adrenal gland	C79.70
Secondary malignant neoplasm of right adrenal gland	C79.71
Secondary malignant neoplasm of left adrenal gland	C79.72
Secondary malignant neoplasm of breast	C79.81
Secondary malignant neoplasm of genital organs	C79.82
Secondary malignant neoplasm of other specified sites	C79.89
Secondary malignant neoplasm of unspecified site	C79.9
Secondary carcinoid tumors, unspecified site	C7B.00
Secondary carcinoid tumors of liver	C7B.02
Secondary carcinoid tumors of bone	C7B.03
Secondary carcinoid tumors of peritoneum	C7B.04
Secondary carcinoid tumors of other sites	C7B.09
Secondary Merkel cell carcinoma	C7B.1
Other secondary neuroendocrine tumors	C7B.8
**Codes used to identify comorbidities in the palliative care algorithm (Table 1)**	
Creutzfeldt-Jakob disease, unspecified	A81.00
Progressive multifocal leukoencephalopathy	A81.2
Vascular dementia, unspecified severity, without behavioral disturbance, psychotic disturbance, mood disturbance, and anxiety	F01.50
Vascular dementia, unspecified severity, with behavioral disturbance	F01.51
Dementia in other diseases classified elsewhere, unspecified severity, without behavioral disturbance, psychotic disturbance, mood disturbance, and anxiety	F02.80
Dementia in other diseases classified elsewhere, unspecified severity, with behavioral disturbance	F02.81
Unspecified dementia, unspecified severity, without behavioral disturbance, psychotic disturbance, mood disturbance, and anxiety	F03.90
Unspecified dementia, unspecified severity, with behavioral disturbance	F03.91
Amnestic disorder due to known physiological condition	F04

© 2025 Parikh RB et al. *JAMA Network Open*.

Systemic atrophy primarily affecting central nervous system in other diseases classified elsewhere	G13.8
Alzheimer's disease with early onset	G30.0
Alzheimer's disease with late onset	G30.1
Other Alzheimer's disease	G30.8
Alzheimer's disease, unspecified	G30.9
Pick's disease	G31.01
Other frontotemporal neurocognitive disorder	G31.09
Senile degeneration of brain, not elsewhere classified	G31.1
Degeneration of nervous system due to alcohol	G31.2
Leigh's disease	G31.82
Neurocognitive disorder with Lewy bodies	G31.83
Corticobasal degeneration	G31.85
Other specified degenerative diseases of nervous system	G31.89
Degenerative disease of nervous system, unspecified	G31.9
Communicating hydrocephalus	G91.0
Obstructive hydrocephalus	G91.1
(Idiopathic) normal pressure hydrocephalus	G91.2
Post-traumatic hydrocephalus, unspecified	G91.3
Other hydrocephalus	G91.8
Hydrocephalus, unspecified	G91.9
Reye's syndrome	G93.7
Unspecified cord compression	G95.20
Other cord compression	G95.29
Unspecified diastolic (congestive) heart failure	I50.30
Acute diastolic (congestive) heart failure	I50.31
Chronic diastolic (congestive) heart failure	I50.32
Acute on chronic diastolic (congestive) heart failure	I50.33
Unspecified combined systolic (congestive) and diastolic (congestive) heart failure	I50.40
Acute combined systolic (congestive) and diastolic (congestive) heart failure	I50.41
Chronic combined systolic (congestive) and diastolic (congestive) heart failure	I50.42

© 2025 Parikh RB et al. *JAMA Network Open*.

Acute on chronic combined systolic (congestive) and diastolic (congestive) heart failure	I50.43
Cerebral infarction, unspecified	I63.9
Chronic obstructive pulmonary disease with (acute) lower respiratory infection	J44.0
Chronic obstructive pulmonary disease with (acute) exacerbation	J44.1
Other specified chronic obstructive pulmonary disease	J44.89
Chronic obstructive pulmonary disease, unspecified	J44.9
Unspecified cirrhosis of liver	K74.60
Chronic kidney disease, stage 1	N18.1
Chronic kidney disease, stage 2 (mild)	N18.2
Chronic kidney disease, stage 3 (moderate)	N18.3
Chronic kidney disease, stage 3 unspecified	N18.30
Chronic kidney disease, stage 3a	N18.31
Chronic kidney disease, stage 3b	N18.32
Chronic kidney disease, stage 4 (severe)	N18.4
Chronic kidney disease, stage 5	N18.5
End stage renal disease	N18.6
Chronic kidney disease, unspecified	N18.9

© 2025 Parikh RB et al. *JAMA Network Open*.

eFigure 3. Sample EHR Inbox Message Sent for Intervention Patients

Subject: re: Eligible for Palliative Care

Dear Dr. _______,

Tennessee Oncology is committed to offering palliative care to patients with advanced cancer.

Mr.____ is eligible for palliative care referral due to having Stage III Esophageal and Esophagogastric Junction Cancer with COPD, ECOG performance score of 2, and age >70.

If you agree with palliative care consultation, there is no need to reply. After 48 hours, we will proceed with calling, introducing and offering palliative care to the patient using a validated script. Your patient will have the opportunity to ask questions about palliative care and can decline referral.

If you DO NOT wish to proceed with palliative care referral, please reply Opt-Out. If you opt-out, please indicate why from the following choices:
[ ] I have already referred to palliative care.
[ ] I wish to discuss this with my patient first.
[ ] I do not believe this patients disease or clinical factors warrant palliative care.
[ ] I worry about the patient getting the wrong message with our treatment goals.
[ ] Other please explain

Sincerely,

Sandhya Mudumbi, MD (Site Principal Investigator)
Stephen Schleicher, MD (Chief Medical Officer)
Natalie Dickson, MD (President and Chief Strategy Officer)

© 2025 Parikh RB et al. *JAMA Network Open*.

eFigure 4. Palliative Care Script

Introducing Palliative Care Study Script

- I'm a nurse from Tennessee Oncology and I work with the supportive and palliative care program. We want to offer this service to you to help improve your quality of life.
- We work together with your Oncologist and while they focus on treating your illness, our team helps manage your symptoms and quality of life.
- A palliative care physician or nurse practitioner will meet with you to review where you are in your journey (illness and treatment), your symptoms and needs, and come up with a plan for those and coordinate any resources that could be helpful. This first meeting usually lasts about 1 hour and you can bring whomever you like. After that, they'll stay involved in your care as needed.
- They serve as an extra support a lot of times for questions that come up and resource for you besides your oncologist.

- When someone is diagnosed with cancer or any serious illness, the fear and uncertainty can be overwhelming. The distress comes in many forms; physical, psychological, social, spiritual, financial. They are here to help you through that distress.
-

FAQ

- **Is there a cost or Is it covered by insurance?** It is covered by insurance just like any other medical specialty like your oncology visits. Depending on your insurance, you may have a copay like your oncology visit.
- **Did the doctor say something was wrong?** I can tell you're worried. This service is actually available to all of our cancer patients. We are offering it to you because of certain factors like having been to the ER/hospital recently, having symptoms that affect your quality of life, distress, or other health issues besides your cancer.
- **I have too many doctor's visits and it'll be hard for me to make it.** I understand that's overwhelming to have so many visits. Our provider can meet you in-person on the same day as your Oncologist or on a treatment day and make the travel easier on you. We can also do telemedicine if you are able to do that.
- **Can my family member come?** Yes, you are welcome to bring anyone that supports you to the visit.
- **How is this different from hospice care?**
  Palliative care is part of your cancer care to help you with quality of life and living well as you go through treatments. Hospice care is focused on comfort for the end of life when a person has decided not to or can't go through more cancer treatment. In palliative care, a person does not have to give up treatment that might cure or treat their cancer and it can be alongside the cancer treatment.

eTable 4. Reasons for Clinician Opt Out

Inappropriate
**Doing too well**
The clinician feels the patient's health and symptom burden are such that palliative care would be unnecessary
[1] "Patient has curative disease, inappropriate for palliative care"
[2] "There is no need, patient has PS of zero without side effect"
[5] "Do not believe this patient's disease or clinical factors warrant palliative care"
[7] "No Medical Intervention-Off Treatment"
[13] "She does have advanced cancer but has been treated definitively and we are optimistic she may achieve a long term remission. She is following at MD Anderson as well. I do not think she needs additional Palliative Care support at this time but will be sure to send her in the future if she develops new Sx related to her cancer. Thank you." (two categories)
[17] "Although on paper he qualifies, due to his targeted therapy, he has no evidence of active dz currently. DWH"
[18] "ok with me pt has some dementia and is doing well she may not see need at this time"
[19] "No need for referral. NO.5, patient feels great, no issue that needs to be addressed."
[21] "i dont think she is a good candidate she is doing great and works part time"
[23] "He has no evidence of active cancer after 10+ years of tarceva therapy. I would defer Palliative care in this patient"
[24] "I think I will pass on palliative referral for her. She has been on Opdivo for 7 years without any evidence of disease."
[27] "He had surgery and had a complete pathologic response - so off therapy and we follow him at this time. Not sure he needs palliative. Happy to discuss further"

© 2025 Parikh RB et al. *JAMA Network Open*.

**Patient/Clinic Factors**
The clinician perceives environmental or personal barriers to the patient seeing palliative care
[4] "Patient is angry and wife is anxious. A phone call at this time would not be good and would be likely to provoke them further."
[10] "Patient currently admitted in the hospital for a nononcologic issue."
[14] "she can but she has transportation problems, is blind, son has to bring on day off, lives in dayton"
[16] "doesn't think patient would want this service"
[22] "He doesn't speak English--son-in-law does. alt."
[31] "OPT out Patient lives in KY WE were told previously to NOT schedule appointments with Med Onc on same day as Palliative CARE as the practice does not get reimbursed appropriately if the patients are seen on the same day by both us an palliative care I try to avoid this although palliative often still schedules on same day as the patient sees oncology"
[15] "i want to hold off i think he is going to hospice very soon"
Timing too early
The clinician does not rule out the possibility of a referral in the future, but does not see it as necessary yet
[8] "Not necessary at this time."
[9] "Does not feel he needs this currently"
[11] "Not necessary at this time."
[12] "no not yet"
[13] "She does have advanced cancer but has been treated definitively and we are optimistic she may achieve a long term remission. She is following at MD Anderson as well. I do not

© 2025 Parikh RB et al. *JAMA Network Open*.

think she needs additional Palliative Care support at this time but will be sure to send her in the future if she develops new Sx related to her cancer. Thank you." (two categories)
[28] "No referral needed at this time"
[29] "Let's hold a palliative care referral for now. Thanks. mw"
Needs discussion first
The clinician would prefer to discuss palliative care directly with the patient before making a referral
[3] "wish to discuss first with patient"
[6] "Worry about the patient getting the wrong message with our treatment goals. I wish to discuss this with my patient first."
[20] "Will discuss with her on Monday. Thank you."
[25] "I would like to discuss with her first. Thank you."
[26] "Will discuss with him at next visit, thank you."
Already receiving palliative care
The patient is already seeing palliative care outside of Tennessee Oncology
[32] "He is seeing Palliative in the community, thank you."
[33] "This patient already follows with Compassus palliative care services at home."
No reason given
The clinician did not provide context to their choice
[30] "no"

© 2025 Parikh RB et al. *JAMA Network Open*.

eTable 5. Characteristics of Patients Opted Out vs Not Opted Out in the Intervention Arm

	Not Opted-out (N=265)	Opted-out (N=31)	Total (N=296)	p value
**Sex**				0.26
Female	134 (50.6%)	12 (38.7%)	146 (49.3%)	
Male	131 (49.4%)	19 (61.3%)	150 (50.7%)	
**Age**				0.24
Mean (SD)	68.11 (10.12)	65.77 (12.33)	67.86 (10.38)	
Median	70.00	68.00	70.00	
**Race**				0.97
White	206 (77.7%)	26 (83.9%)	232 (78.4%)	
Black or African American	34 (12.8%)	3 (9.7%)	37 (12.5%)	
Asian	7 (2.6%)	1 (3.2%)	8 (2.7%)	
Hispanic	2 (0.8%)	0 (0.0%)	2 (0.7%)	
Native American	1 (0.4%)	0 (0.0%)	1 (0.3%)	
Other	2 (0.8%)	0 (0.0%)	2 (0.7%)	
Unknown/Unspecified	13 (4.9%)	1 (3.2%)	14 (4.7%)	
**Ethnicity**				0.06
Hispanic	5 (1.9%)	0 (0.0%)	5 (1.7%)	
Not Hispanic	195 (73.6%)	17 (54.8%)	212 (71.6%)	

© 2025 Parikh RB et al. *JAMA Network Open*.

	Not Opted-out (N=265)	Opted-out (N=31)	Total (N=296)	p value
Unknown/Unspecified	65 (24.5%)	14 (45.2%)	79 (26.7%)	
**Diagnosis**				0.82
Lung Malignancies	206 (77.7%)	25 (80.6%)	231 (78.0%)	
Non-Colorectal GI Malignancies	59 (22.3%)	6 (19.4%)	65 (22.0%)	
**Stage**				0.84
Stage III	80 (30.2%)	10 (32.3%)	90 (30.4%)	
Stage IV	185 (69.8%)	21 (67.7%)	206 (69.6%)	
**Metastatic**				0.50
Metastatic	204 (77.0%)	22 (71.0%)	226 (76.4%)	
Non-metastatic	61 (23.0%)	9 (29.0%)	70 (23.6%)	
**Risk Score**				0.78
N	265	31	296	
Mean (SD)	3.05 (2.84)	2.90 (2.60)	3.03 (2.81)	
Median	2.00	2.00	2.00	

© 2025 Parikh RB et al. *JAMA Network Open*.

eTable 6. Reasons Cited by Patients for Declining Palliative Care Referral

Patient doesn't have any active symptoms and does not feel there is a need for palliative care.
Pt states Oncologist does a good job managing my symptoms right now and they are being controlled.
Pt stated this was not discussed with Oncologist and would like to wait and discuss with him/her.
Pt stated they are very overwhelmed right now with all the appointments and would like to wait and see if our services are needed.
Pt already sees pain management, so they don't need to see us too.
Pt relies on family members to bring them to appointments, and they don't want to burden them with another appt. Pt uncomfortable using TM or speaking over the phone.

© 2025 Parikh RB et al. *JAMA Network Open*.

eTable 7. Adjusted Changes in Palliative Care Referral Rates in Secondary Analyses

	Unadjusted palliative care rates (%)		Adjusted hazard ratio (95% CI)	p-value[c]
Population	Control (n=266)	Intervention (n=296)		
Overall	8.3	43.9	6.9 (3.7, 12.7)	<0.001
**Subgroup analyses**				
Sex				0.86
Female	9.4	48.0	6.6 (2.7, 16.0)	
Male	7.3	40.0	7.1 (3.7, 13.5)	
Age[a]				0.09
≤ 70 yr	10.3	46.6	5.9 (2.8, 12.7)	
> 70 yr	5.8	40.7	9.2 (4.4, 19.0)	
Race/Ethnicity[a]				0.03
Non-Hispanic White	9.4	41.3	5.6 (3.0, 10.3)	
Other[b]	3.9	53.0	19.6 (4.8, 80.8)	
Malignancy				0.002
Lung	5.5	43.3	10.8 (4.7, 24.4)	
Non-Colorectal GI	17.2	46.2	3.3 (1.9, 5.8)	
Stage				0.55
III	6.5	45.6	8.7 (3.1, 24.4)	

© 2025 Parikh RB et al. *JAMA Network Open*.

IV	9.3	43.2	6.0 (2.7, 13.4)	
Risk score				0.44
≤3	7.8	43.6	7.0 (3.2, 15.4)	
> 3	9.5	44.9	6.3 (3.2, 12.5)	

NOTE: Secondary analyses used Cox proportional hazards models, adjusting for patient age, sex, diagnosis, stage, and risk score using robust standard errors to account for clustering by clinic. We derived adjusted hazard ratios for 12-week palliative care rates from Cox proportional hazards models. Hazard ratios >1 indicate greater effect on palliative care for intervention relative to control.

[a]Race, ethnicity, and sex were taken from the EHR, which is primarily captured via patient self-report

[b]Other = Hispanic, Black or African American, Asian, Native American, Unknown/Unspecified

[c]P-value for the primary analysis in the overall cohort comes from the adjusted Cox model, using α=0.05 to define statistical significance. p-value for the 6 subgroup analyses reflect significance of intervention-by-subgroup interaction terms, using α=0.05 to define statistical significance.

© 2025 Parikh RB et al. *JAMA Network Open*.

eTable 8. Adjusted Changes in Patient-Reported Metrics Not Using the Carry-Forward Method

	Control (N=83 completed baseline PAL-14 assessment; N=85 completed baseline Heard and Understood assessment)		Intervention (N=73 completed baseline PAL-14 assessment and baseline Heard and Understood assessment)		Mean adjusted difference between intervention and control (95% CI)	p-value
	Number (%) completing followup survey	Mean score difference	Number (%) completing followup survey	Mean score difference		
Mean change in PAL-14 between 0 and 4 weeks (SD)	72 (86.7)	1.03 (5.34)	72 (98.6)	1.09 (5.99)	0.18 (0.01, 0.37)	0.49
Mean change in PAL-14 between 0 and 12 weeks (SD)	61 (73.5)	2.08 (5.73)	67 (91.8)	1.72 (6.74)	-0.32 (-0.51, -0.18)	<0.001
Mean change in PAL-14 between 0 and 24 weeks (SD)	40 (48.2)	0.88 (5.49)	38 (52.1)	0.82 (7.97)	-0.02 (-0.56, 0.52)	0.83
Mean change in heard and understood between 0 and 4 weeks (SD)	74 (87.1)	-0.11 (0.65)	64 (87.7)	-0.16 (0.56)	-0.05 (-0.11, 0.00)	0.07
Mean change in heard and understood between 0 and 12 weeks (SD)	62 (72.9)	-0.02 (0.67)	67 (91.8)	-0.07 (0.63)	-0.06 (-0.09, -0.02)	0.03
Mean change in heard and understood between 0 and 24 weeks (SD)	40 (47.1)	-0.09 (0.59)	38 (52.1)	-0.13 (0.54)	-0.07 (-0.13, -0.02)	0.19

© 2025 Parikh RB et al. *JAMA Network Open*.

eFigure 5. Overall Survival Kaplan-Meier Plot

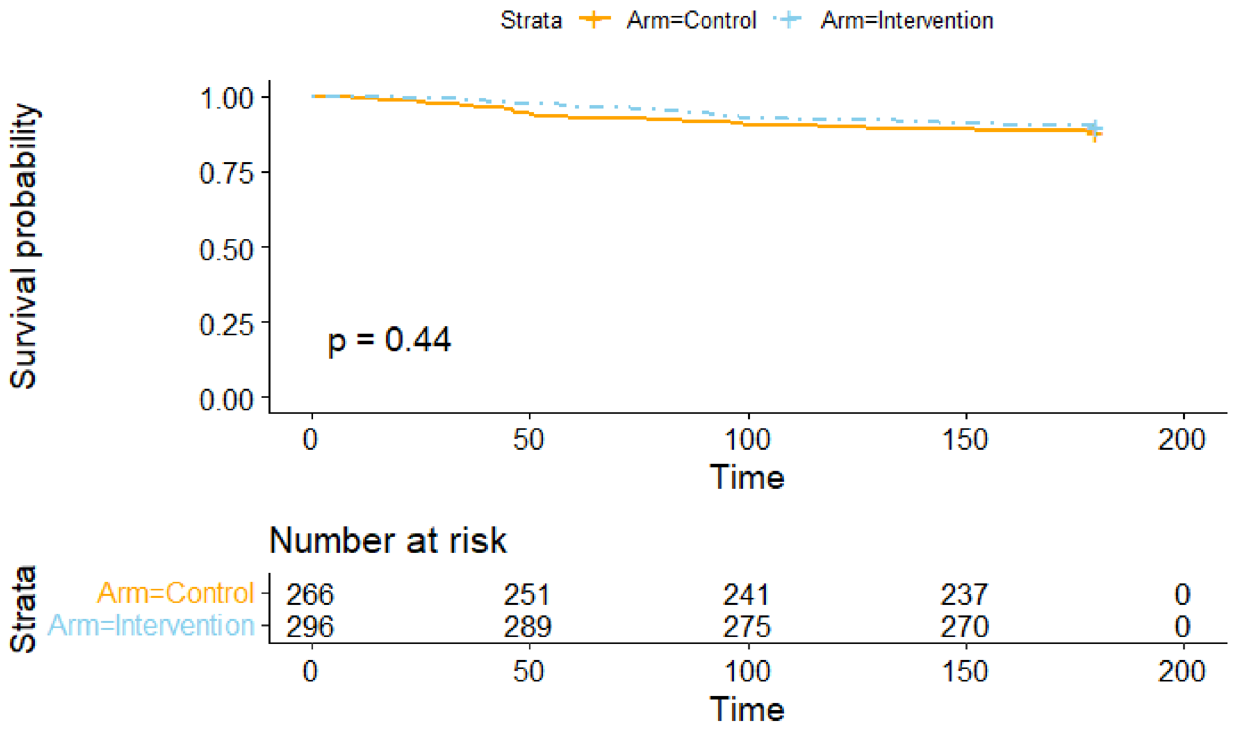

eMethods.

Baseline Clinic Environment

The community oncology network has a large outpatient PC program where a nurse practitioner or physician is embedded within medical oncology clinics. In anticipation of the study and increased volume, the medical director for the PC team ensured adequate capacity for new patients from the study.

Cohort Selection

Stage III and IV lung and non-colorectal cancers were identified using algorithms based on International Classification of Diseases (ICD) diagnosis codes, EHR entries, and manual screening by research staff (**eTable 3**). Patients who did not have an eligible score could develop higher scores in subsequent weeks and subsequently become eligible for the trial. We chose to focus on lung and non-colorectal GI malignancies because these malignancies had a large volume of evidence from randomized trials in support of early PC and were priorities for PC by practice leadership.[1,5]

Algorithm Development

Due to this being a real-world, pragmatic application of an evidence-based practice, our algorithm had to include variables that were easy to collect and incorporate in the electronic health record and were clinically meaningful. We began with the “Indications” section of 2022 National Comprehensive Cancer Network Palliative Care guidelines. We then held a stakeholder meeting with key leaders (medical and radiation oncologists, PC clinicians, nursing and care coordination leaders, data analytics team, billing and coding experts, and operational leaders). This stakeholder group identified data variables that would not be feasible to incorporate into the algorithm due to data abstraction hurdles and also added some clinically

relevant criteria despite not being in the guidelines. For example, we eliminated variables such as prognostic awareness, spiritual concerns, delirium, as we did not have an easy method of abstracting this data from our EHR. While not listed in the guidelines, our stakeholders felt PC would be very beneficial for older adults (age $\geq$ 70) and younger patients (age $\leq$ 40) who may have more severe emotional distress. Additionally, value-based cancer care is a priority at the practice, and while this is not explicit in the NCCN guidelines, we chose to incorporate emergency room visits and hospitalizations and prioritize this by assigning extra points (2 points) to each occurrence of acute care utilization. The pain score was removed as our palliative and care coordination and nursing teams did not find it to be a very reliable measure. We also had difficulty incorporating specific patient-report symptom measures (other than the NCCN distress score) due to those being in a different platform where data could not easily be incorporated into our algorithm.

Depression and Distress Screening

Two of the components of the PC eligibility algorithm (see Table 1) were the NCCN Distress Score and PHQ-2 screen for depression. Distress/Depression screenings were given to every patient in treatment at every physician visit in routine practice. This screening was built into cancer regimens, so that when a physicians orders a regimen in the EHR for a patient, these screens attached to the planned MD visits were already populated. This was what triggered the screening to be given to the patient at each physician visit while they are being treated. We used a proprietary patient communication system to deliver the questionnaires electronically to patients when they checked-in for their physician appt and a screening was due. This was filled out electronically on a tablet directly in the patient communication system. Data was collected in discreet data fields from the questionnaire and databased. Each week, the practice informatics team pulled all patients with a distress screening from the database with their

answers to the distress and depression questions. We used that week's generated list of patients and pulled the medical record numbers (MRNs, primary key between systems) from that list and filtered the distress screening list by those MRNs. We joined the 2 lists together by the MRNs by importing the scores for each pt using the logic "1=yes" or "0=no", where "Yes" means they screened positive per our algorithm (≥8 for distress and ≥3 PHQ-2) and no means they did not screen positive. Points were calculated in the overall risk score.

Randomization

We stratified randomization by clinic volume to avoid the possibility of large clinics being disproportionately randomized to the intervention, which may have resulted in a large number of specialty PC referrals and strained capacity. Prior to trial initiation, we grouped each of the 15 clinics into 8 pairs of practices, based on volume of patients that would have been identified by the algorithm in the past 24 weeks. Clinics were then assigned unique identifiers to maintain blinding and randomized 1:1 to the intervention vs. control using a random number generator in a stratified fashion, such that one clinic in each group was represented in either intervention or control arm in mutually exclusive fashion (**eTable 2** in Supplement 1). The smallest-volume clinic was randomly assigned to one of the arms, such that 8 clinics were represented in 1 arm and 7 clinics in the other.

Triaging palliative care referrals

In the intervention arm, urgency of palliative care consultation depended on risk score. For patients with risk score above 8, they were attempted to be scheduled within 2 weeks. All other patients were attempted to be scheduled within 4 weeks.

Recruitment for and administration of surveys

Because we wished to compare the impact of algorithm-driven PC on patient-reported metrics, we assessed the quality of life and heard and understood metrics only among specific subsets of patients in each arm (see **eMethods**). Patient-reported metrics were administered by a trained research coordinator by telephone. In the intervention arm, only patients who completed PC visits were offered patient-reported outcome assessments at the first PC visit and at 4-week intervals thereafter. In the control arm, to identify patients unexposed to palliative care, a randomly-selected subset of 5 patients were invited at enrollment to complete patient-reported assessments. If a patient randomized from the control arm did not answer the initial phone call, another patient in the control condition was randomly sampled with replacement; for this reason, the number of control patients randomized to surveys (n=131) was greater than 24 x 5 = 120. We were unable to approach all patients randomized to the control condition for surveys due to limited time availability of research coordinators to approach all patients for surveys. Among patients who completed a baseline assessment, absolute change in FACIT-PAL 14 and Heard and Understood scores between baseline and 4, 12, and 24 weeks were compared between intervention and control arms. Of note, patients were required to provide informed consent in order to report survey outcomes.
