# Supplementary material for: Algorithm-Based Palliative Care in Patients With Cancer: A Cluster Randomized Clinical Trial
Source: JAMA Netw Open. 2025 Feb 21;8(2):e2458576. doi: 10.1001/jamanetworkopen.2024.58576 (PMC11846008; doi:10.1001/jamanetworkopen.2024.58576)
Supplement: Supplement 2. — Trial Protocol [file jamanetwopen-e2458576-s002.pdf]

TITLE PAGE

Protocol Title: Predictive Analytics and Behavioral Nudges to Improve Palliative Care in Advanced Cancer

BE A PAL*: Behavioral Economics and Analytics to improve PALliative care in advanced cancer*

Protocol Number: #851021

Amendment Number: 7

Version Number: 7

Sponsor: Tennessee Oncology, PLLC

Approval Date: Version 1 approved April 2022

CONFIDENTIAL

The information contained in this document, especially any unpublished data, is the property of Tennessee Oncology, PLLC (or under its control) and therefore is provided to you in confidence as an investigator, potential investigator, or consultant, for review by you, your staff, and an applicable Ethics Committee or Institutional Review Board. It is understood that this information will not be disclosed to others without written authorization from Tennessee Oncology, PLLC except to the extent necessary to obtain informed consent from persons whom participate.

TABLE OF CONTENTS

1. Synopsis
2. Background
   - 2.1 Advanced Cancer Burden
   - 2.2 Palliative Care Improves Quality of Life & Symptoms
   - 2.3 Palliative Care Rarely Used
   - 2.4 Improve Short-Term Mortality Prediction
   - 2.5 Palliative Care Underutilized
   - 2.6 Palliative Care Biases
   - 2.7 Palliative Care Constraints
   - 2.8 Palliative Care Utilization Improvements
3. OBJECTIVES AND OUTCOMES
   - 3.1 Overall Objectives
   - 3.2 Primary Outcome
   - 3.3 Secondary Outcomes
4. STUDY DESIGN
   - 4.1 Randomization & Recruitment
   - 4.2 Potential Study Benefits
   - 4.3 Study Duration
   - 4.4 Target Population
   - 4.5 Inclusions/Exclusions
   - 4.6 Interventions
   - 4.7 Innovations
   - 4.8 Cohort Malignancies
   - 4.9 Accrual
   - 4.10 Qualitative Procedures
   - 4.11. Subject Compensation
5. ASSESSMENTS
   - 5.1 Risk Benefit
6. STATISTICAL CONSDERATIONS AND ANAYLSIS PLAN
   - 6.1 Generalized Considerations
   - 6.2 Patient & Family Perceptions
   - 6.3 Risk Score
   - 6.4 Analysis Measures
   - 6.5 Sample Size
   - 6.6 Risks & Benefits
   - 6.7 Investigators
7. DATA COLLECTION AND MANAGEMENT
   - 7.1 Data Quality Assurance
   - 7.2 Data Disclosure
   - 7.3 Data Safety & Monitoring
   - 7.4 Source Data Documentation
   - 7.5 Use of Computerized Systems
   - 7.6 Retention of Records
8. ETHICAL CONSIDERATIONS
   - 8.1 Compliance with Laws & Regulations
   - 8.2 Review Boards & Ethics Committee
   - 8.3 Informed Consent
   - 8.4 Confidentiality
9. STUDY DOCUMENTATION, MONITORING, AND ADMINISTRATION
   - 9.1 Study Documentation
   - 9.2 Protocol Deviations
   - 9.3 Clinic Inspections

9.4 Administrative Structure
9.5 Publication of Data and Protection of Trade Secrets
9.6 Protocol Amendments
10. REFERENCES

LIST OF EXHIBITS

- Exhibit 1. Predictive Risk Score
- Exhibit 2. Trial Schema
- Exhibit 3. Organizational Structure
- Exhibit 4. Patient Surveys
- Exhibit 5. EHR Nudge
- Exhibit 6. Recruitment Emails for Oncology and Palliative Care Clinicians
- Exhibit 7. VERBAL CONSENT FORM

1. SYNOPSIS

Patients with advanced cancer suffer from high symptom burden and aggressive end-of-life care. Early specialty palliative care is an evidence-based practice that improves symptom burden, quality of life, and survival in advanced cancer. However, over half of patients with advanced cancer die before receiving palliative care. Clinician-level biases and suboptimal identification of high-risk patients are major barriers to palliative care uptake. In this 2-arm pragmatic clinical trial, we will randomize practices within a large community oncology network to receive an intervention consisting of algorithm-based default palliative care referrals. We will study the impact of such an intervention on palliative care utilization and end-of-life outcomes.

2. BACKGROUND

2.1 ADVANCED CANCER BURDEN

Over half of patients with advanced cancer report moderate-to-severe symptom burden and poor quality of life – both of which are associated with up to 70% lower overall survival.[1–3] Despite heavy symptom burden, 40% of patients with advanced cancer receive aggressive end-of-life care, including chemotherapy and lack of hospice referral close to death, that is not concordant with patient goals.[4] Suboptimal symptom management, poor communication about expected treatment benefit, and lack of attention to patient goals and wishes near the end of life contribute to these gaps.[5]

2.2 PALLIATIVE CARE IMPROVES QUALITY OF LIFE & SYMPTOMS

Palliative care is a medical specialty focused on providing relief from the symptoms and stress of serious illnesses such as cancer and is available in inpatient, outpatient, and community-based settings.[6] Outpatient palliative care is available at 98% of NCI-designated cancer centers and 63% of non-NCI centers.[7] Early outpatient palliative care concurrent with cancer-directed treatment improves quality of life, reduces symptom burden, and decreases rates of aggressive end-of-life care.[8,9] Since 2017, the American Society of Clinical Oncology has recommended specialty outpatient palliative care consultation for patients within 8 weeks of advanced cancer diagnosis.[10] During the COVID-19 pandemic, other organizations have called for earlier palliative care to ensure that high-risk cancer care meets patients' goals.[11,12] Despite such guidelines, nearly two-thirds of patients with advanced cancer do not receive palliative care prior to death.[4] Delayed or missed outpatient palliative care referrals are a major contributor to aggressive end-of-life care.[8]

2.3 PALLIATIVE CARE RARELY USED

NCCN-based indications for palliative care referral include limited prognosis and prognostic risk factors, such as uncontrolled symptoms or poor performance status.[13] Better awareness of mortality risk may inform clinicians' decisions around palliative care referral and prompt goal-concordant cancer care.[14] However, oncologists correctly identify only 20% of patients with advanced cancer who will die in one year and overestimate prognosis for 70% of patients.[15,16] Furthermore, existing palliative care triggers ignore patient- and cancer-specific heterogeneity in important variables such as laboratories and comorbidities.[17]

2.4 IMPROVE SHORT-TERM MORTALITY PREDICTION

Advances in electronic health record (EHR) infrastructure and predictive analytics allow accurate and automated identification of patients with cancer at risk of short-term mortality. We have trained and deployed EHR-based predictive algorithms with better performance (c-statistic >0.80; sensitivity >60%) than traditional prognostic aids into routine oncology practice in order to identify patients who may benefit from early palliative care and advance care planning.[18,19] At Tennessee Oncology, a rules-based automated EHR algorithm based on 14 components derived from 2021 NCCN guidelines **(Exhibit 1)** accurately identifies patients at risk of 180-day-month mortality.[20] This algorithm has been incorporated in pilot studies, and has generated weekly reports of high-risk patients who may benefit from timely palliative care referral.

<u>There is an urgent need to implement strategies based on algorithm-based triggers to increase early outpatient palliative care among patients with advanced cancer.</u>

2.5 PALLIATIVE CARE UNDERUTILIZED

Two-thirds of patients with advanced cancer do not receive palliative care prior to dying. Furthermore, clinicians underutilize palliative care, usually initiating referrals only 2 months before death. Lack of standardized referral and screening criteria for outpatient palliative care contributes to underutilization. This is particularly true for Black and Hispanic populations, for whom palliative care referrals are 50% lower compared to White populations.

2.6 PALLIATIVE CARE BIASES

S*tatus quo bias*, which predisposes clinicians to continue current practice even if not the optimal option, may lead to delayed or missed palliative care referrals. Additionally, *optimism bias,* the cognitive bias that causes clinicians to believe that their own patients are at lesser risk of negative outcomes, may cause clinicians to underestimate a patient's mortality risk, thus delaying palliative care referral. Finally, *overconfidence bias,* the propensity to overestimate one's desired behaviors when it is not objectively reasonable, may lead clinicians to incorrectly believe they are initiating similar or more palliative care referrals than their peers.

2.7 PALLIATIVE CARE CONSTRAINTS

Despite increasing availability in tertiary cancer care settings, specialty palliative care is sparsely available in community oncology practices – where 75% of patients receive their primary oncologic care. Furthermore, while the number of patients with cancer eligible for palliative care is expected to grow by 20% in the upcoming decade, there will be a shortage of 18,000 palliative care specialty physicians, particularly in the outpatient setting. Because of these capacity constraints, it is crucial to identify scalable <u>strategies to automatically identify high-risk patients with advanced cancer in order to initiate timely outpatient palliative care referrals.</u>

2.8 PALLIATIVE CARE UTILIZATION IMPROVEMENTS

Overcoming suboptimal clinician decision-making biases is key to increasing palliative care referrals. Principles from behavioral economics can inform “nudges” that change how clinicians receive information and make choices such as palliative care referral. Default, opt-out nudges that make the optimal choice the path of least resistance can mitigate clinicians’ status quo bias. Reframing clinicians’ prognoses via “triggered” identification of high-risk patients may combat optimism bias. Finally, peer comparisons use social comparisons to counter overconfidence bias in order to increase guideline-based practice. These strategies are associated with 10-25 absolute percentage-point increases in guideline-based practices such as statin prescribing and transition from brand to generic drugs. However, to our knowledge no published randomized trials have used behavioral strategies to improve palliative care utilization in advanced cancer.

<u>Given rising demand for palliative care with constrained supply across the United States oncology care system, our contribution will be significant because it will leverage scalable automated predictive algorithms with a behaviorally informed intervention to increase palliative care utilization among high-risk patients with advanced cancer. This intervention is expected to create a feasible, adaptable, and acceptable process in a community oncology setting that increases palliative care utilization earlier in the advanced cancer disease trajectory.</u>

3. OBJECTIVES & OUTCOMES

3.1 OVERALL OBJECTIVES

Our main objective is to evaluate the impact of an intervention consisting of default algorithm-based referrals, compared to usual practice, on outpatient palliative care visits and quality of end-of-life care among patients with advanced cancer.

We will also explore physician and practice-related facilitators and barriers to completion via mixed-methods interviews. After completing initial enrollment, we aim to conduct a qualitative study through approximately 20 semi-structured interviews with:

1. medical oncology clinicians in the intervention group to understand the impact of the risk-algorithm and behavioral nudges on their workflow, and explore barriers and facilitators for feasibility, scalability, and ultimately increasing palliative care referrals for patients with advanced lung or non-colorectal gastrointestinal malignancies.
2. palliative care clinicians who saw patients referred through the study to explore their perspectives on seeing patients identified by the risk-algorithm and default referrals.

3.2 PRIMARY OUTCOME

Completed palliative care visit within 3 months among high-risk patients with stage III and IV lung and non-colorectal GI malignancies

3.3 SECONDARY OUTCOMES

3.4 EXPLORATORY OUTCOMES

We will also assess on an exploratory basis the impact of the intervention on:

- Aggressive end-of-life care via hospice enrollment (≤3 days before death)
- Aggressive end-of-life care via systemic therapy (receipt within 14 days of death)
- Change in quality of life of patients from baseline to 24 weeks, via the FACIT-PAL 14
- Change in feeling "Heard & Understood" instrument from baseline to 24 weeks (see **Exhibit 4**).

4. STUDY DESIGN

4.1 RANDOMIZATION & RECRUITMENT

This 2-arm randomized trial (six-month enrollment; six-month follow-up) will enroll patients with advanced lung and gastrointestinal malignancies and 40 clinicians to assess response to behavioral nudges to refer to palliative care at up to 17 practices within Tennessee Oncology, a US community oncology network. All practices have access to palliative care and will be randomized to usual care vs. intervention. The outcome is completion of a palliative care visit within 3 months among high-risk patients.

We will randomize practices stratified by clinic volume to avoid the possibility of large clinics being disproportionately randomized to the intervention, which may result in a large number of specialty PC referrals and strained capacity (see **Exhibit 1**). The Principal Investigator (RBP) and primary statistical analyst will be blinded to arm assignment.

This 2-arm randomized trial (six-month enrollment, six-month follow-up) is taking place at Tennessee Oncology. Tennessee Oncology, one of the nation's largest, community-based cancer care specialists, is home to one of the leading clinical trial networks in the country.

Eligible clinicians will receive notification about the trial via email and in-person presentations. High-risk patients for the intervention will be identified from weekly runs of the risk score algorithm, in coordination with Larry Bilbrey, Tennessee Oncology Care Data Systems Manager.

For the semi-structured interviews, the research team at Tennessee Oncology will create a list of potential clinicians. We will initially contact potential participants via email (see **Exhibit 6**), and if we do not get enough participation, coordinator and site investigator will do in-person recruitment with clinicians. For participants that agree to do the interview, clinical research coordinator will create a research ID for each participant that only key members of research team will have access to. Coordinator will subsequently create a de-identified dashboard to track mode of interview (in-person, virtual, telephone), length of interview, participant characteristics (gender, years in practice, palliative care v oncology, physician v nurse practitioner), and to store audio files, transcripts, and analysis documents. To ensure an adequate number of perspectives on the trial, we will use purposive sampling to sample clinicians who performed at or above vs. below the median response to the intervention.

4.2 POTENTIAL STUDY BENEFITS

Potential study benefits include improved access to early palliative care and end-of-life care outcomes in patients with advanced cancer.

4.3 STUDY DURATION

- 12 months (6 months enrollment; 6 months follow-up). Of note, enrollment will continue for the entire 6 month enrollment period.
- We anticipate the semi-structured interviews take approximately three to four months. In a qualitative study, interviews are conducted until thematic saturation is achieved, and we anticipate this will be achieved with interviewing approximately 10 oncology clinicians in the intervention group and 10 palliative care clinicians.

4.4 TARGET POPULATION

Patients with advanced malignancies and their clinicians, to assess response to behavioral nudges to refer to palliative care at up to 17 practices within Tennessee Oncology. The split will be into two arms: intervention vs. control, with up to 40 clinicians in each arm. Clinicians will be clustered to a single practice for the intervention and analysis in order to avoid contamination of intervention effect into the usual care arm.

Our target population for the semi-structured interviews includes clinicians at Tennessee Oncology that were included either in the intervention group as oncology providers, and palliative care clinicians at Tennessee Oncology that saw patients referred through our intervention.

4.5 INCLUSIONS/EXCLUSIONS

Eligible clinicians will be medical oncology physicians and advance practice providers (APPs) practicing at Tennessee Oncology. Clinicians will be clustered to a single practice for the intervention and analysis in-order-to avoid contamination of intervention effect into the usual care arm. Patients will be excluded if they had a palliative care visit within the past 90 days or had no medical oncology visit within the prior 24 weeks months or are seen for a non-medical oncology encounter.

Eligible clinicians will receive notification about the trial via email and in-person presentations. High-risk patients for the intervention will be identified from weekly runs of the risk score algorithm (see **Exhibit 1**), in coordination with Tennessee Oncology Care Data Systems Manager.

Practicing medical oncology care clinicians that were in the intervention arm of our initial study and practicing palliative care clinicians at Tennessee Oncology are eligible for the semi-structured interviews. Medical oncology clinicians at Tennessee Oncology that were in control arm will be excluded.

<u>Inclusion Criteria</u>

- Clinicians:
    - Medical oncology physicians and advance practice providers (APPs) practicing at Tennessee Oncology
- Patients:
    - Stage III and IV lung, and non-Colorectal GI cancers, defined using internal algorithms based on International Classification of Diseases (ICD) diagnosis codes, EHR entries, and manual screening

- Received cancer treatment (chemotherapy or immunotherapy) in the past 6 months

Exclusion Criteria

- Patients:
  - Benign hematology, genetics, survivorship encounters;
  - No prior EHR data;
  - Deceased or enrolled in hospice care;
  - Had a palliative care visit within the prior three months;
  - Have not had a medical oncology visit within the prior 6 months or are seen for a non-medical oncology encounter

4.6 INTERVENTIONS

Clinicians of patients in both arms will receive education on the availability of early palliative care and performance reports. Clinicians in Arm 1 (intervention) will receive a EHR notification with option to opt-out (placed by the research coordinator, see **Exhibit 5**) for palliative care referral for any eligible high-risk patient, defined by a risk score ≥1 for Stage IV cancer patients and ≥2 for Stage III cancer patients. If the risk score is above 8, they will be scheduled within 2 weeks, and all other patients will be scheduled within 4 weeks. Clinician will have the option to opt-out for any patient by responding to the notification which will be sent to the research coordinator. If the clinician does not respond, the research coordinator will approach patient via telephone, explain the rationale for referral based on a predetermined script, and offer and schedule an outpatient or telemedicine palliative care consultation per patient preference. Follow-up visits will occur at the discretion of the palliative care clinician, usually monthly. Clinicians in both arms will receive monthly peer comparison emails that compare their palliative care referral rates against other clinicians.

4.7 INNOVATIONS

Prior retrospective studies have analyzed the effect of educational or trigger-based strategies on palliative care utilization. However, three factors limit generalizability: (1) lack of a prospective, randomized design; (2) academic study settings with different patient populations and palliative care capacity than most US oncology practices; and (3) referral triggers that require substantial clinician cognitive effort. Our approach of a pragmatic randomized clinical trial is unique. Furthermore, our behavioral intervention, using default referrals based on an automated EHR algorithm, tests a scalable approach to increasing palliative care utilization. Finally, we will use post-intervention semi-structured interviews to refine this approach prior to submitting an R01 application for a multi-institutional randomized clinical trial that includes algorithm-based behavioral strategies to increase palliative care referral in community oncology.

4.8 COHORT MALIGNANCIES

Stage III and IV lung and non-colorectal Gastrointestinal (GI) cancers, defined using internal algorithms based on administrative codes, EHR entries, and treatment regimens. These malignancies represent 43% of solid tumor deaths in the US, have positive prospective evidence for early outpatient palliative care, and have been used in prior Tennessee Oncology palliative care pilots.

4.9 ACCRUAL

Patients will accrue to the trial as their clinic or clinician in enrolled. Included Tennessee Oncology practices will be randomly assigned to usual care or intervention groups. When enrollment starts, clinicians in the intervention clinics will receive the EHR nudge for palliative care referral for each eligible patient and based on the clinician response (opt-out or default), the research coordinator will call and offer palliative care consultation visit to the patient.

For the semi-structured interviews, participants will be initially recruited via internal practice email (See **Exhibit 6**) from the clinical research coordinator. If we don't receive enough interest, we will then recruit by visiting the clinicians in-person in their clinics. In-person recruitment will be done by site investigator and/or clinical research coordinator at Tennessee Oncology.

Approximately 20 eligible clinician participants from the intervention group will be interviewed (1) in-person, (2) virtually, or (3) via telephone. To ensure an adequate number of perspectives on the trial, we will use purposive sampling to sample clinicians who performed at or above vs. below the median response to the intervention. The clinical research coordinator will email and schedule the interviews through outlook. A confirmation email will be sent to the clinician 24 hours before the interview unless the interview will occur less than 24 hours after scheduling. Two pilot interviews will be conducted and used in analysis (pending major edits are not required).

4.10 QUALITATIVE PROCEDURES

1. Interviews will be conducted in-person or virtually at a time and location that is convenient for the participant. Typically, this would be in their home clinic within their private office space. If needed, there is office space available in our practice administrative suite in Nashville, TN. If it is difficult to schedule an in-person interview, or if the interviewer is the Penn CRC, we will offer a virtual (zoom or skype) interview or telephone interview.
2. There will be two CRCs conducting interviews for this study. One of the CRCs is based at Tennessee Oncology and one is based at Penn. Both will be trained by the research team on conducting semi-structured interviews.
3. All interview supplies will be readied before the interview begins. The following items are needed: recorder and charger, laptop and charger, interview guide.
4. At time of interview, clinical research coordinator (interviewer) will follow the listed procedures in order:
    a. A final confirmation will be made that the clinician can talk at that time
    b. The interviewer will confirm the email or address to which compensation should be sent after the interview.
    c. Interviewer will obtain verbal consent using the consent language included in the appendix.
    d. Interviewer will begin recording on audio recording device or Zoom. Interviewer will state clinician ID number and date of the interview.
    e. Interviewer will conduct semi-structured interview based on a prepared interview guide with open-ended questions either in-person, over Zoom, or on the telephone.

f. To wrap up interview, the interviewer will ask if the clinicians has any final thoughts or questions. Once these are addressed, interviewer will state that recording will stop, and the interviewer will stop the audio recorder.
g. At the end of the interview, the interviewer will debrief the participant. This is not a structured process, but rather time to answer any questions the participant has about the study. We don’t anticipate significant enough emotional distress in clinical providers to warrant support resources.

5. Immediately following the end of the call, the interviewer will save the audio recording on a password-protected laptop, upload to Monday.com study dashboard, and memo the interview by writing down an overall summary of the interview and if there is anything else that was noteworthy. This document will be saved for future use as needed in Monday.com.
6. The interviewer will also send a brief thank you note and incentive via email.

Post-Interviews

1. As the interviews are being completed, audio recordings will be collected and sent for transcription by a third-party transcription company. When files are sent to the external company, the staff member will note to the transcribers to remove any individual names of people (e.g., clinicians, health system leaders, staff, patients), sites, etc. that have been used in transcript if any are mentioned during recording.
2. After transcription has been completed, the coding and analysis process will begin.

Coding Interviews

1. Interview coding will require a minimum two individuals (this can include the interviewing research staff member)
2. Both coders will carefully read each interview and then meet to discuss common themes
3. Once coders have discussed common themes, a list of coding nodes will be created for use in Dedoose qualitative software.
4. One coder will import the transcripts and insert the node list in a single file. Both coders will use this same origin file as they code.
5. The coders will meet every 2-3 interviews that they code to discuss their coding and to ensure that there are few to no discrepancies between coding styles.
6. The two coders will each code all transcripts.
7. The codebook will be edited on an ongoing basis as needed, as determined by the emergence of new themes.
8. Following the completion of successful coding, Dedoose can be used to pull different qualitative data points as needed for analysis.
9. Coders will develop theme sheets based on those excerpts identified in coding as relevant to each theme
10. The team will review theme sheets and discuss the message of each theme.
11. The team will come to consensus on quotes that are exemplary of each theme.

4.11 SUBJECT COMPENSATION

We will offer participants in the semi-structured interviews monetary compensation of $100 with a Clincard gift card.

5. ASSESSMENTS

5.1 RISK BENEFIT

The risk/benefit ratio of this study is very favorable. The study team will take necessary steps to maintain confidentiality and privacy throughout the study period.

The potential risks associated with this study are minimal. Breach of data is a potential risk that will be mitigated by using HIPAA compliant and secure data platforms as described. There are also potential emotional risks to patients if they misunderstand the role of palliative care. Our research coordinator will be trained in helping patients and their caregivers understand the supportive role of palliative care.

There may be some emotional discomfort or distress with doing patient-reported surveys. If this occurs, subjects will have the option to stop the phone call and questionnaire, and we will also provide support resources to help cope with this afterwards including referral to our internal psychology team or state-funded mental health support crisis hotline (855-274-7471).

6. STATISTICAL CONSIDERATIONS AND ANALYSIS PLAN

6.1 GENERALIZED CONSIDERATIONS

Our study setting is a large community-based oncology network. This population is similar to many other community oncology practices in that it is not affiliated with a large hospital or health system, and thus the major source of palliative care is in the outpatient setting. However, Tennessee Oncology has a robust outpatient and telemedicine palliative care infrastructure consisting of 2.5 FTE physicians and 8.0 FTE advance practice providers. Our study findings may be challenging to generalize to oncology practices with limited access to outpatient specialty palliative care. However, within the next 5-10 years, most community oncology practices will have access to ambulatory or telemedicine outpatient palliative care. Furthermore, our algorithm-based approach accounts for limited palliative care capacity by customizing thresholds; thus, it is more flexible than previously-used rules-based triggers.

We expect to generate results and manuscripts within 2 years due to quick enrollment and the presence of an existing and accepted risk score, which obviates the need to create another algorithm. If necessary, we will submit an amended version requirement by spacing out PI, co-investigator, and research coordinator effort and enrollment over a three-year period and by using a higher risk score threshold to control high-risk patient enrollment.

Due to payer considerations with the COVID-19 pandemic, telemedicine palliative care has been reimbursed at ambulatory rates; thus, 40% of palliative care visits at Tennessee Oncology are currently via telemedicine. Reimbursement landscape changes may impact palliative care access,

although this would occur in both the control and intervention arms and should not compromise the intervention effect.

6.2 PATIENT & FAMILY PERCEPTIONS

The phrase “palliative care” is often stigmatized to the point where patients and their caregivers sometimes are fearful of this clinical service, worried that it means cancer-directed treatment is no longer effective. Our coordinator will educate and explain the focus on quality of life regardless of their treatment plan or outcome. Furthermore, we will make every effort to schedule in-person or telemedicine visits to minimize patient and caregiver burden

6.3 RISK SCORE

To identify patients who may benefit most from early outpatient palliative care, we use a rules-based algorithm based on 14 NCCN-endorsed criteria for palliative care referral. This stands in contrast to previous randomized trials that have randomized patients to palliative care at the point of diagnosis. Compared to such trials, our approach may identify patients relatively late and does not pre-specify the frequency of palliative care appointments. However, this approach is generalizable to other systems that collect similar data, since outpatient specialty palliative care at the point of diagnosis is often infeasible due to lack of palliative care capacity. Furthermore, our algorithm still identifies patients well before the immediate end-of-life period, and thus is expected to increase referrals among many eligible patients. The risk algorithm also ensures that the highest risk patients get seen earlier and in a more-timely fashion.

6.4 ANALYSIS MEASURES

The primary study endpoint is completion of a palliative care visit within 3 months of enrollment *among high-risk patients*.

We will also assess on an exploratory basis the impact of the intervention on:

- Aggressive end-of-life care via hospice enrollment (≤3 days before death)
- Aggressive end-of-life care via systemic therapy (receipt within 14 days of death)
- Change in quality of life of patients from baseline to 24 weeks, via the FACIT-PAL 14
- Change in feeling “Heard & Understood” instrument from baseline to 24 weeks (see **Exhibit 4**).

Patients will be followed for exploratory outcomes for six months or death.

The semi-structured interviews will be transcribed and uploaded to Atlas.ti, a data management software. We will inductively develop a codebook following the initial interviews based on the structure of the interview guide. One of two reviewers will code each transcript, and approximately 20-25% of total transcripts will be coded by both reviewers to establish inter-rater reliability. The adequacy of the codebook will be periodically assessed by the reviewers in partnership with the research team and modifications will be made as their understanding of the data and emergent themes evolves over time. The reviewers will meet regularly to discuss discrepancies and update the code book as needed, with record keeping adequate to track changes to the code book and the

rationale. Kappa statistics will be generated to estimate inter-rater reliability. Coding will then be reviewed to summarize key themes, and representative quotes will be selected to illustrate those themes.

6.5 SAMPLE SIZE

Currently, there are approximately 4000 eligible patient-encounters over a six-month period at our study practices. Given prior work showing the algorithm flags 8-10% of eligible patients as high-risk, we anticipate enrolling at least 360 patients. Of note, enrollment will continue past 360 patients until we reach the end of the six-month enrollment period. The unit of analysis is the patient. We will use multilevel mixed-effects models, adjusted for relevant covariates, to assess outcomes. Assuming baseline palliative care rate=26.9% (SD=10%), a 10% dropout rate, two-sided alpha=0.05, a sample of 360 patients (180 intervention, 180 control) will provide 80% power to detect a meaningful 17 percentage-point increase in palliative care visits.

6.6 RISKS & BENEFITS

The risk/benefit ratio of this study is very favorable. The study team will take necessary steps to maintain confidentiality and privacy throughout the study period.

The potential risks associated with this study are minimal. Breach of data is a potential risk that will be mitigated by using HIPAA compliant and secure data platforms as described. There are also potential emotional risks to patients if they misunderstand the role of palliative care. Our research coordinator will be trained in helping patients and their caregivers understand the supportive role of palliative care.

There may be some emotional discomfort or distress with doing patient-reported surveys. If this occurs, subjects will have the option to stop the phone call and questionnaire, and we will also provide support resources to help cope with this afterwards including referral to our internal psychology team or state-funded mental health support crisis hotline (855-274-7471).

Potential study benefits include improved consideration of stakeholder input into our planned palliative care intervention and more informed decision-making regarding palliative care treatment plans.

6.7 INVESTIGATORS

Ravi Parikh, MD, MPP is the Principal Investigator. Dr. Parikh is an Assistant Professor in the Department of Medical Ethics and Health Policy and Medicine at the University of Pennsylvania with experience implementing pragmatic clinical trials of similar scale at the University of Pennsylvania Health System.

Sandhya Mudumbi, MD is the Principal Investigator. Dr. Mudumbi is Medical Director of Palliative Care at Tennessee Oncology, a multispecialty network of community oncology practices in Tennessee and Northern Georgia.

Stephen Schleicher, MD, MBA is Chief Medical Officer and Medical Oncologist at Tennessee Oncology, PLLC. (See **Exhibit 3**)

7. DATA COLLECTION AND MANAGEMENT

7.1 DATA QUALITY ASSURANCE

Tennessee Oncology will supply Electronic Medical Record (EMR) specifications for this study. Tennessee Oncology will be responsible for data management of this study, including quality checking of the data. Data entered manually will be collected via EMR using source documents. Clinics will be responsible for data entry into the EMR system. In the event of discrepant data, Tennessee Oncology will request data clarification from the clinics to resolve electronically in the Electronic Data Capture (EDC) system.

Tennessee Oncology will produce a Data Quality Plan that describes the quality checking to be performed on the data. EMR and correction documentation will be maintained in the EDC system's audit trail. System backups for data stored at the clinic(s) and records retention for the study data will be consistent with the Tennessee Oncology standard procedures.

Tennessee Oncology will perform oversight of the data management of this study, including approval of data management plans and specifications. Tennessee Oncology standard procedures will be used to handle and process the electronic transfer of these data.

7.2 DATA DISCLOSURE

Information will not be disclosed to anyone outside of the study team.

7.3 DATA SAFETY AND MONITORING

All data will be stored on secured Tennessee Oncology servers and all files will be password protected.

7.4 SOURCE DATA DOCUMENTATION

Tennessee Oncology monitor(s) will perform ongoing source data verification to confirm that critical protocol data (i.e., source data) entered into the Electronic Medical Record (EMR) by authorized site personnel are accurate, complete, and verifiable from source documents.

Source documents (paper or electronic) are those in which patient data are recorded and documented for the first time. They include, but are not limited to, hospital records, clinical and office charts, laboratory notes, memoranda, patient-reported outcomes, evaluation checklists, pharmacy dispensing records, recorded data from automated instruments, copies of transcriptions that are certified after verification as being accurate and complete, microfiche, photographic negatives, microfilm or magnetic media, radiology reports, patient files, and records kept at pharmacies, laboratories, and medico-technical departments involved in a clinical trial.

Before study initiation, the types of source documents that are to be generated will be clearly defined in the Trial Monitoring Plan. This includes any protocol data to be entered directly into the EMR (i.e., no prior written or electronic record of the data) and considered source data.

Source documents that are required to verify the validity and completeness of data entered into the EMR must not be obliterated or destroyed and must be retained per the policy for retention of records described in Section **7.6.**

To facilitate source data verification, the investigators and institutions must provide University of Pennsylvania direct access to applicable source documents and reports for trial-related monitoring, audits, and IRB/EC review. The clinics must also allow inspection by applicable health authorities.

7.5 USE OF COMPUTERIZED SYSTEMS

When clinical observations are entered directly into the clinic's computerized medical record system (i.e., in lieu of original hardcopy records), the electronic record can serve as the source document if the system has been validated in accordance with health authority requirements pertaining to computerized systems used in clinical research. An acceptable computerized data collection system allows preservation of the original entry of data. If original data are modified, the system should maintain a viewable audit trail that shows the original data as well as the reason for the change, name of the person making the change, and date of the change.

All Tennessee Oncology users and any use of any IT resource(s) are required to read and understand our data transmission security policy and procedure.

The purpose of our Data Transmission Security Policy and Procedures is to guard against unauthorized access to electronic protected health information that is being transmitted over an electronic communications network. The establishment and implementation of effective Data Transmission Security Procedures is a crucial element in our overall objective or providing reasonable protections for individually identifiable health information, including Protected Health Information ("PHI", as defined by HIPAA).

7.6 RETENTION OF RECORDS

Records and documents pertaining to the conduct of this study and the distribution of data, including EMR, paper PRO data (if applicable), Informed Consent Forms, laboratory test results, and medication inventory records must be retained by the Principal Investigator for at least 15 years after completion or discontinuation of the study, or for the length of time required by relevant national or local health authorities, whichever is longer. After that period of time, the documents may be destroyed, subject to local regulations.

The research team will destroy audio-recordings 18 months after the study is complete. All data will be de-identified when stored. We will destroy all data 18 months after the study is complete.

No records may be disposed of without the written approval of Tennessee Oncology. Written notification should be provided to Tennessee Oncology prior to transferring any records to another party or moving them to another location.

8. ETHICAL CONSIDERATIONS

8.1 COMPLIANCE WITH LAWS AND REGULATIONS

This study will be conducted in full conformance with the ICH E6 guideline for Good Clinical Practice and the principles of the Declaration of Helsinki, or the laws and regulations of the country in which the research is conducted, whichever affords the greater protection to the individual. The study will comply with the requirements of the ICH E2A guideline (Clinical Safety Data Management: Definitions and Standards for Expedited Reporting).

8.2 REVIEW BOARDS & ETHICS COMMITTEE

As required by local regulations, the Investigator will ensure approval of the appropriate regulatory bodies is obtained, prior to study initiation. If required, the Investigator will also ensure that the implementation of substantial amendment to the protocol and other relevant study documents happen only after approval by the relevant regulatory authorities.

The Advarra Institutional Review Board (IRB) will review Tennessee Oncology's research activities. The Advarra IRB is an independent IRB that provides services for academic and non-academic institutions. The study protocol will also be submitted to the University of Pennsylvania IRB for review.

8.3 INFORMED CONSENT

For administering quality of life and communication questionnaires, we request a verbal consent process. This is requested since our intervention will be being conducted via telephone and with the large geographic distribution of our practice (over 130 miles between clinics), it would not be feasible to obtain written consent for the questionnaire component of the study for patients. An information form will be read to the patient and mailed to them if they consent to completing the questionnaire via telephone.

For the intervention itself, a waiver of informed consent is requested. This is a health system initiative that will be implemented. The study is to evaluate that initiative. Therefore, physicians and their patients will not be consented as this is the standard of practice per the health system initiative. Without a waiver of the consent, the initiative would still be implemented by the health system, but the study would be infeasible. There are several additional reasons why we feel a waiver of consent should be granted. First, it is not feasible to consent every physician and as mentioned this initiative would occur with or without the study of it. Second, if members of the control group were consented, this alone could change their behavior. This could potentially disrupt the design of the study and making interpretation of the findings challenging. Third, physicians are not being forced to refer patients to palliative care. This intervention decreases the cognitive burden of remembering which patients are appropriate and would benefit from palliative care. The oncologist always retains control of the referral by being able to “opt-out” and cancel the referral on a case by case basis. This intervention is working towards better implementation of what is considered standard of care for patients with advanced care.

Verbal consent will be obtained from each clinician who participates in the semi-structured interviews (see **Exhibit 7**). Written consent is not necessary for this part of the project because clinicians will not be required to participate and can opt out at any point. No patient identifying information will be provided to clinicians.

8.4 CONFIDENTIALITY

Tennessee Oncology maintains confidentiality standards by coding each patient enrolled in the study through assignment of a unique patient identification number. This means that patient names are not included in data sets that may be transmitted.

All efforts will be made by study staff to ensure subject privacy. Data will be evaluated in a de-identified manner whenever possible. All transcripts will be de-identified and stored in password protected files on a secure folder at Tennessee Oncology.

Computer-based files will only be made available to personnel involved in the study using access privileges and passwords. Wherever feasible, identifiers will be removed from study-related information. Precautions are already in place to ensure the data are secure by using passwords and HIPAA-compliant encryption.

Patient medical information obtained by this study is confidential and may only be disclosed to Tennessee Oncology trained study staff. Electronic data will be stored on secure, password-protected firewalled servers.

Data generated by this study must be available for inspection upon request by representatives of the U.S. FDA and other national and local health authorities, representatives, and collaborators, and the IRB/EC for each study site, as appropriate.

The research team at the University of Pennsylvania will not have access to identifiable information in this trial. All data for this project will be stored on the secure/firewalled servers of the DART system, in data files that will be protected by multiple password layers. These data servers are maintained in a guarded facility behind several locked doors, with very limited physical access rights. They are also cyber-protected by extensive firewalls and multiple layers of communication encryption. Electronic access rights are carefully controlled by University of Pennsylvania system managers. We believe this multi-layer system of data security, identical to the system protecting the University of Pennsylvania Health Systems medical records, greatly minimizes the risk of loss of privacy. In addition, risk of loss of confidentiality will be minimized by storing completed paper copies of the surveys and signed informed consent forms in locked file cabinets in locked offices accessible only to trained study staff. Each subject will be assigned a unique identifier without identifying information, and data will be entered into an electronic database using only the unique identifier. Only trained study staff will have access to the code that links the unique identifier to the subject's identity. Electronic data will be stored on secure, password-protected firewalled servers at the University of Pennsylvania.

9. STUDY DOCUMENTATION, MONITORING, AND ADMINISTRATION

9.1 STUDY DOCUMENTATION

The investigator must maintain adequate and accurate records to enable the conduct of the study to be fully documented, including but not limited to the protocol, protocol amendments, and documentation of IRB/EC and governmental approval when applicable. In addition, at the end of the study the investigation will receive the patient data, which includes an audit trail containing a complete record of all changes to data.

9.2 PROTOCOL DEVIATIONS

The investigator should document and explain any protocol deviations. The investigator should promptly report any deviations that might impact study patient data integrity to the IRB/EC in accordance with established IRB/EC policies. As per Tennessee Oncology's standard operating

procedures, perspective to deviate from the protocol, including requests to waive protocol eligibility criteria are not allowed.

9.3 CLINIC INSPECTIONS

Clinic visits will be conducted by Tennessee Oncology or an authorized representative for inspection of study data, patients' medical records, and source documentation. The investigator will permit national and local authorities, collaborators, and the IRBs/ECs to inspect facilities and records relevant to this study.

9.4 ADMINISTRATIVE STRUCTURE

The study is sponsored by Tennessee Oncology, PLLC and University of Pennsylvania. Tennessee Oncology Research Team will perform study and safety monitoring, data collection and management. Data analysis and dissemination of study findings including publication will be in collaboration with research team led by Dr. Ravi Parikh at University of Pennsylvania.

9.5 PUBLICATION OF DATA AND PROTECTION OF TRADE SECRETS

Regardless of the outcome of a trial, Tennessee Oncology is dedicated to openly providing information on the trial to healthcare professionals and to the public, both at scientific congresses and in peer-reviewed journals. Tennessee Oncology will comply with all requirements for publication of study results.

In accordance with standard editorial and ethical practice, Tennessee Oncology will support publication of multi-centers only in their entirety and not as an individual center data.

9.6 PROTOCOL AMENDMENTS

Any protocol amendments will be prepared by Tennessee Oncology. Protocol amendments will be submitted to the IRB/IEC and to regulatory authorities in accordance with local regulatory authorities.

A protocol amendment will be provided if data inclusion/exclusion criteria has been added to the study.

Approval will be obtained from the IRB/IEC and regulatory authorities (as locally required) before implementation of any changes, except for changes necessary to eliminate an immediate hazard to the patients or changes that involve logistical or administrative aspects only.

10. <u>REFERENCES</u>

1. Nipp RD, El-Jawahri A, Moran SM, et al. The relationship between physical and psychological symptoms and health care utilization in hospitalized patients with advanced cancer. *Cancer*. 2017;123(23):4720-4727. doi:10.1002/cncr.30912

2. Hwang SS, Chang VT, Fairclough DL, Cogswell J, Kasimis B. Longitudinal Quality of Life in Advanced Cancer Patients: Pilot Study Results from a VA Medical Cancer Center. *Journal of Pain and Symptom Management*. 2003;25(3):225-235. doi:10.1016/S0885-3924(02)00641-3

3. Subbiah IM, Charone MM, Roszik J, et al. Association of Edmonton Symptom Assessment System Global Distress Score With Overall Survival in Patients With Advanced Cancer. *JAMA Network Open*. 2021;4(7):e2117295. doi:10.1001/jamanetworkopen.2021.17295

4. Langton JM, Blanch B, Drew AK, Haas M, Ingham JM, Pearson S-A. Retrospective studies of end-of-life resource utilization and costs in cancer care using health administrative data: a systematic review. *Palliat Med*. 2014;28(10):1167-1196. doi:10.1177/0269216314533813

5. Sullivan DR, Chan B, Lapidus JA, et al. Association of Early Palliative Care Use With Survival and Place of Death Among Patients With Advanced Lung Cancer Receiving Care in the Veterans Health Administration. *JAMA Oncology*. 2019;5(12):1702-1709. doi:10.1001/jamaoncol.2019.3105

6. Palliative Care Definition | What is Palliative Care. Accessed January 17, 2021. https://www.capc.org/about/palliative-care/

7. Hui D, De La Rosa A, Bruera E. State of Integration of Palliative Care at National Cancer Institute–Designated and Nondesignated Cancer Centers. *JAMA Oncol*. 2020;6(8):1292-1295. doi:10.1001/jamaoncol.2020.1471

8. Temel JS, Greer JA, Muzikansky A, et al. Early palliative care for patients with metastatic non-small-cell lung cancer. *The New England Journal of Medicine*. 2010;363(8):733-742. doi:10.1056/NEJMoa1000678

9. Zimmermann C, Swami N, Krzyzanowska M, et al. Early palliative care for patients with advanced cancer: a cluster-randomised controlled trial. *Lancet (London, England)*. 2014;383(9930):1721-1730. doi:10.1016/S0140-6736(13)62416-2

10. Ferrell BR, Temel JS, Temin S, et al. Integration of Palliative Care Into Standard Oncology Care: American Society of Clinical Oncology Clinical Practice Guideline Update. *JCO*. 2017;35(1):96-112. doi:10.1200/JCO.2016.70.1474

11. Cavalier JS, Maguire JM, Kamal AH. Beyond Traditional Advance Care Planning: Tailored Preparedness for COVID-19. *Journal of Pain and Symptom Management*. 2020;0(0). doi:10.1016/j.jpainsymman.2020.08.020

12. Curtis JR, Kross EK, Stapleton RD. The Importance of Addressing Advance Care Planning and Decisions About Do-Not-Resuscitate Orders During Novel Coronavirus 2019 (COVID-19). *JAMA*. 2020;323(18):1771-1772. doi:10.1001/jama.2020.4894

13. Levy M, Smith T, Alvarez-Perez A, et al. Palliative Care Version 1.2016. *J Natl Compr Canc Netw*. 2016;14(1):82-113. doi:10.6004/jnccn.2016.0009

14. Finlay E, Casarett D. Making difficult discussions easier: using prognosis to facilitate transitions to hospice. *CA Cancer J Clin*. 2009;59(4):250-263. doi:10.3322/caac.20022

15. Christakis NA, Smith JL, Parkes CM, Lamont EB. Extent and determinants of error in doctors' prognoses in terminally ill patients: prospective cohort studyCommentary: Why do doctors overestimate?Commentary: Prognoses should be based on proved indices not intuition. *BMJ*. 2000;320(7233):469-473. doi:10.1136/bmj.320.7233.469

16. Chow E, Harth T, Hruby G, Finkelstein J, Wu J, Danjoux C. How accurate are physicians' clinical predictions of survival and the available prognostic tools in estimating survival times in terminally ill cancer patients? A systematic review. *Clin Oncol (R Coll Radiol)*. 2001;13(3):209-218.

17. Kamal AH, Bull J, Kavalieratos D, Taylor DH, Downey W, Abernethy AP. Palliative Care Needs of Patients With Cancer Living in the Community. *J Oncol Pract*. 2011;7(6):382-388. doi:10.1200/JOP.2011.000455

18. Manz CR, Chen J, Liu M, et al. Validation of a Machine Learning Algorithm to Predict 180-Day Mortality for Outpatients With Cancer. *JAMA oncology*. Published online September 24, 2020. doi:10.1001/jamaoncol.2020.4331

19. Manz CR, Parikh RB, Small DS, et al. Effect of Integrating Machine Learning Mortality Estimates With Behavioral Nudges to Clinicians on Serious Illness Conversations Among Patients With Cancer: A Stepped-Wedge Cluster Randomized Clinical Trial. *JAMA Oncol*. Published online October 15, 2020:e204759. doi:10.1001/jamaoncol.2020.4759

20. Dans M, Kutner JS, Agarwal R, et al. NCCN Guidelines® Insights: Palliative Care, Version 2.2021. *J Natl Compr Canc Netw*. 2021;19(7):780-788. doi:10.6004/jnccn.2021.0033

EXHIBIT 1

EXHIBIT 1 – Risk Algorithm

Risk Factor	Point Assignment Rule	Factor Value	Risk Score Point
Bone Metastases in Diagnosis List or Charge	If exists	1	1
Secondary malignant neoplasm of brain	If exists	1	1
COPD in diagnosis list or charge	If exists	1	1
CHF in diagnosis list or charge	If exists	1	1
Dementia in Diagnosis List or Charge	If exists	1	1
Liver Cirrhosis in diagnosis list or charge	If exists	1	1
Stroke in diagnosis list or charge	If exists	1	1
Spinal cord compression in Diagnosis list or charge	If exists	1	1
Most recent NCCN Distress Score	≥8	1	1
Most recent ECOG Performance Score	≥2	1	1
Most recent PHQ-2 Score	≥3	1	1
Number of ED visits in prior 3 months	Each ED visit receives 2 points	1	2
Number of Inpatient stays in prior 3 months	Each Inpatient stay receives 2 points	1	2
Low Age	≤40	1	1
High Age	≥70	1	1

Total Risk Score	Interpretation	Time to Palliative Care Visit
≥1 Stage IV, ≥2 Stage III	High Risk	2 weeks
<1 Stage IV, <2 Stage III	Non-High Risk	4 weeks

EXHIBIT 2

Exhibit 2. Trial Schema

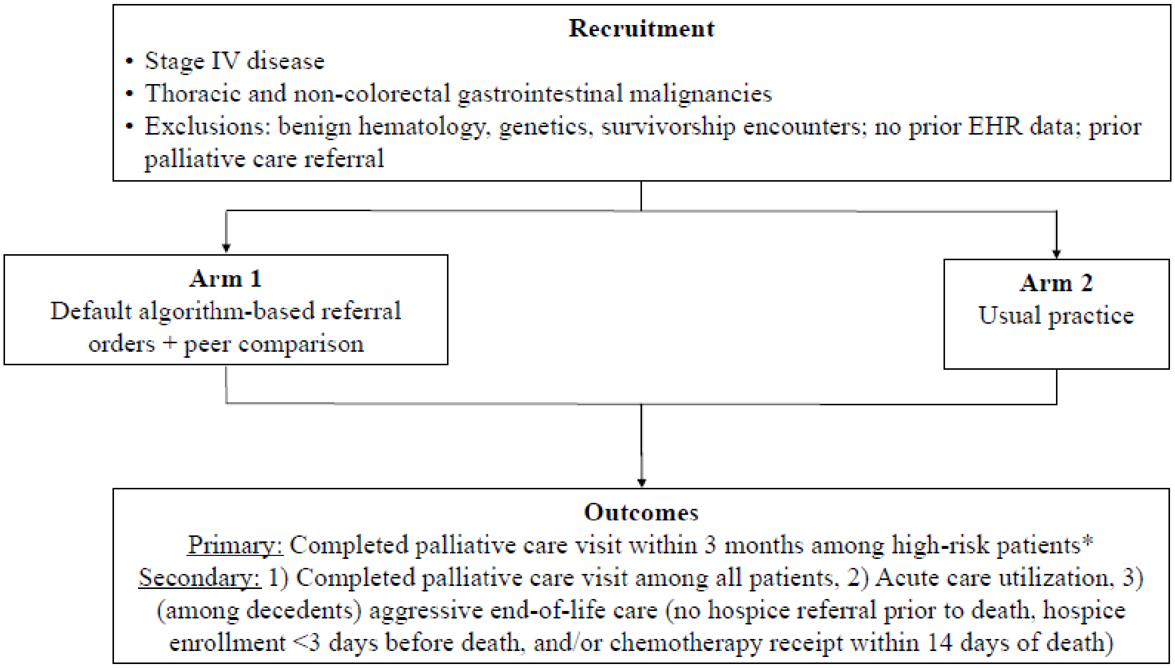

*Score ≥1 Stage IV, ≥2 Stage III – high risk on algorithm

EXHIBIT 3

Exhibit 3. Organizational Structure

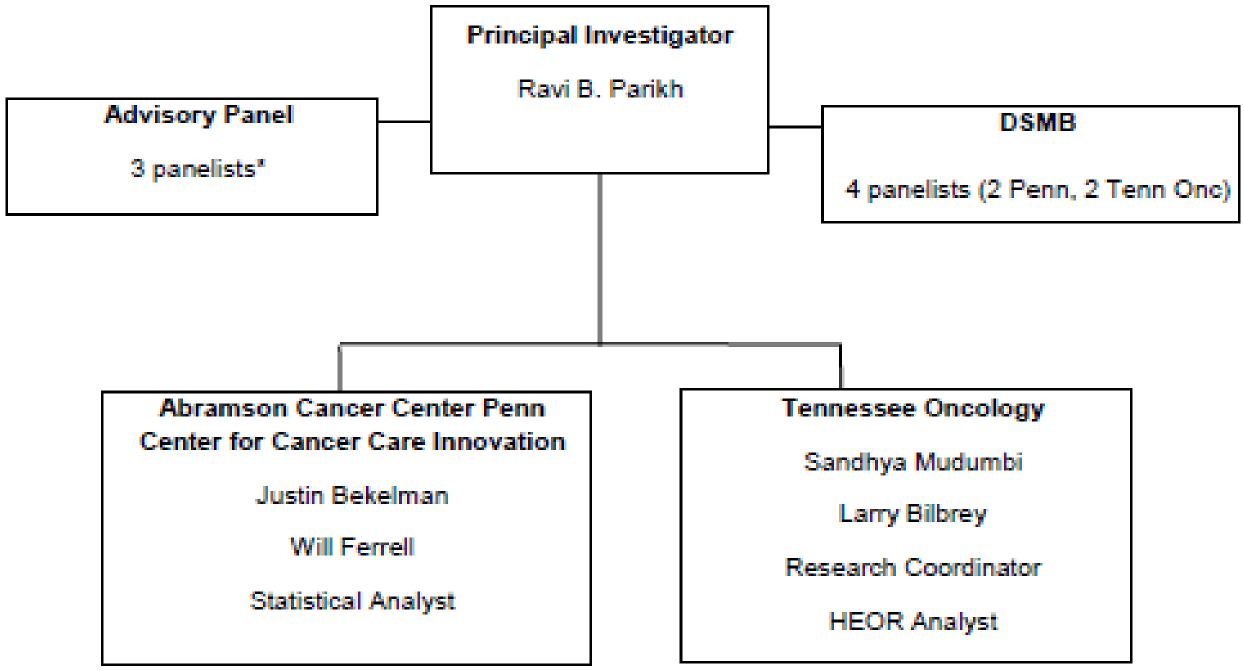

*Panelists with expertise in machine learning, informatics, palliative care, and cancer care delivery. As per NIH guidance, we do not name panel members in this application so as not to preclude reviewers from their academic institutions.

EXHIBIT 4

Patient Surveys

FEELING HEARD AND UNDERSTOOD

Individuals can respond to each data element using discrete values ranging from 1 (Completely true) to 5 (Not at all true.)

Q1. I felt heard and understood by this provider and team.				
1. Completely true	2. Very true	3. Somewhat true.	4. A little bit true.	5. Not at all true.

Q2. I felt this provider and team put my best interests first when making recommendations about my care.				
1. Completely true	2. Very true	3. Somewhat true.	4. A little bit true.	5. Not at all true.

Q3. I felt this provider and team saw me as a person, not just someone with a medical problem.				
1. Completely true	2. Very true	3. Somewhat true.	4. A little bit true.	5. Not at all true.

Q4. I felt this provider and team understood what is important to me in my life.				
1. Completely true	2. Very true	3. Somewhat true.	4. A little bit true.	5. Not at all true.

FACIT-PAL 14

Below is a list of statements that other people with your illness have said are important**. Please circle or mark one number per line to indicate your response as it applies to the past 7 days.**

		Not at all	A little bit	Some-what	Quite a bit	Very much
GP1	I have a lack of energy	0	1	2	3	4
GP2	I have nausea	0	1	2	3	4
GP4	I have pain	0	1	2	3	4
GE6	I worry that my condition will get worse	0	1	2	3	4
GF3	I am able to enjoy life	0	1	2	3	4
GF5	I am sleeping well	0	1	2	3	4
GF7	I am content with the quality of my life right now	0	1	2	3	4
GS2	I get emotional support from my family	0	1	2	3	4
Sp21	I feel hopeful	0	1	2	3	4
GE1	I feel sad	0	1	2	3	4
Pal4	I feel like a burden to my family	0	1	2	3	4
Pal5	I am constipated	0	1	2	3	4
Pal14	I am able to openly discuss my concerns with the people closest to me	0	1	2	3	4
B1	I have been short of breath	0	1	2	3	4

EXHIBIT 5

EHR notification with option to opt-out

Subject: Patient eligible for palliative care referral

Tennessee Oncology is committed to offering palliative care to patients with advanced cancer.

Mr./Mrs____ is eligible for palliative care referral due to _____________ (include risk factors that flagged them here).

If you agree with palliative care consultation, there is no need to reply. After 48 hours, we will proceed with calling, introducing and offering palliative care to the patient using a validated script. Your patient will have the opportunity to ask questions about palliative care and to decline.

If you DO NOT wish to proceed with palliative care referral, please reply "Opt-Out". If you opt-out, please indicate why from the following choices:

[ ] I have already referred to palliative care.

[ ] I wish to discuss this with my patient first.

[ ] I do not believe this patient's disease or clinical factors warrant palliative care.

[ ] I worry about the patient getting the wrong message with our treatment goals.

[ ] Other – please explain

Sincerely,

Sandhya Mudumbi, MD

Stephen Schleicher, MD

Natalie Dickson, MD

EXHIBIT 6

Recruitment Emails for Oncology and Palliative Care Clinicians

Recruitment Email for Oncology Clinicians

Subject: Re: your feedback on upenn palliative care study

Dear Dr. ___ or NP name,

We have completed enrollment for our palliative care clinical trial with University of Pennsylvania and would love your **feedback on being a participant in the intervention group.**

This is a separate qualitative study where you will be interviewed, and your feedback will be incorporated to improve our model for palliative care referrals among patients with advanced lung and non-colorectal GI malignancies.

We are offering a $100 gift card for your time and participation.

Let me know when a good time for us to meet and conduct the interview for approximately 30 minutes. I can meet with you in person (for middle TN), virtually (zoom or skype), or via telephone.

Thank you for your time and consideration.

Sincerely,
Nikki Johnson, RN

Recruitment Email for Palliative Care Clinicians

Subject: Re: your feedback on upenn palliative care study

Dear Dr. ___ or NP name,

We have completed enrollment for our palliative care clinical trial with University of Pennsylvania and would love your **feedback on being a palliative care clinician in this study.**

This is a separate qualitative study where you will be interviewed, and your feedback will be incorporated to improve our model for palliative care referrals among patients with advanced lung and non-colorectal GI malignancies.

We are offering a $100 gift card for your time and participation.

Let me know when a good time for us to meet and conduct the interview for approximately 30 minutes. I can meet with you in person (for middle TN), virtually (zoom or skype), or via telephone.

Thank you for your time and consideration.

Sincerely,
Nikki Johnson, RN

EXHIBIT 7

VERBAL CONSENT FORM

Title of Research: Clinician Perspectives on Predictive Analytics and Behavioral Nudges to Improve Palliative Care in Advanced Cancer

Principal Investigator: Ravi Parikh, MD
Site Investigator: Sandhya Mudumbi, MD

Sponsor: University of Pennsylvania, Tennessee Oncology

Purpose of the Research

We are asking you to take part in an interview to hear your perspectives as a participant in a clinical trial. The trial aimed to increase utilization of Tennessee Oncology's embedded palliative care service in advanced lung and non-colorectal GI cancer patients through an "opt-out" nudge or alert. You've been asked to participate in this interview because you were a part of this study, either as an Oncology or as a palliative care provider.

Explanation of Procedures

If you agree, I will ask that you to participate in a 30-45 minute interview and share with us your perspective on this trial, and barriers and facilitators to scaling this model to other Oncology practices.

This interview will be audio-recorded, transcribed, and analyzed to incorporate your feedback to improve this model of palliative care referrals.

Benefits

Participating in this interview by giving your honest feedback will give you an active role in developing this model.

Risks and Discomforts

There are very minimal risks to participating in this interview. The main potential risk is a breach of confidentiality; however, your name and information will be not be shared

Confidentiality

We want to ensure that you feel comfortable sharing your honest opinions and feedback. If you choose to participate, you will be assigned a unique ID number. The link between your name and ID number will be kept in a separate database that is accessible only to key study personnel. Everything you say today will be kept confidential. The recordings will be destroyed at the completion of the study. Any identifying information, like your name or your clinic, will be removed from that transcript.

Voluntary Participation and Withdrawal

You do not have to answer any questions that you do not want to. You may withdraw from the interview at any time by simply asking to stop your participation.

Cost of Participation

The cost of participating is your time, which we greatly appreciate and for which we offer a token of appreciation.

<u>Payment for Participation in Research</u>

You will receive $100 after your interview, even if you choose to withdraw.

<u>Questions</u>

If you have any questions, concerns, or complaints about the research or a research-related injury including available treatments, please contact the principal investigator, Dr. Ravi Parikh, or the site investigator, Dr. Sandhya Mudumbi at 615-202-7797.

If you have questions about your rights as a research participant, or concerns or complaints about the research, you may contact Advarra IRB at *410.884.2900.*

We are obtaining verbal consent for your participation in this interview, and therefore if you agree, we will move forward with the interview. If you would like, a copy of this consent form can be emailed or mailed to you.

Printed Name of Person Obtaining Consent

Signature of Person Obtaining Consent and Date

Reviewed by:

Printed Name of Principal Investigator

Signature of Principal Investigator and Date
