# Supplementary material for: Algorithm-Based Palliative Care in Patients With Cancer: A Cluster Randomized Clinical Trial
Source: JAMA Netw Open. 2025 Feb 21;8(2):e2458576. doi: 10.1001/jamanetworkopen.2024.58576 (PMC11846008; doi:10.1001/jamanetworkopen.2024.58576)
Supplement: Supplement 3. — Data Sharing Statement [file jamanetwopen-e2458576-s003.pdf]

Data Sharing Statement

Parikh. Algorithm-Based Palliative Care in Patients With Cancer. *JAMA Netw Open*. Published February 10, 2025. doi:10.1001/jamanetworkopen.2024.58576

Data
Additional Information: ClinicalTrials.gov: NCT05590962
Data available: Yes
Data types: Deidentified participant data, Data dictionary
How to access data: Requests can be sent to Ravi.Bharat.parikh@emory.edu
When available: With publication

Supporting Documents
Document types: Statistical/analytic code, Informed consent form
How to access documents: Requests can be sent to Ravi.Bharat.parikh@emory.edu
When available: With publication

Additional Information
Who can access the data: researchers whose proposed use of the data has been approved
Types of analyses: For any purpose
Mechanisms of data availability: after approval of a proposal, with a signed data access agreement
